# HBeeID: a molecular tool that identifies honey bee subspecies from different geographic populations

**DOI:** 10.1186/s12859-024-05776-9

**Published:** 2024-08-27

**Authors:** Ravikiran Donthu, Jose A. P. Marcelino, Rosanna Giordano, Yudong Tao, Everett Weber, Arian Avalos, Mark Band, Tatsiana Akraiko, Shu-Ching Chen, Maria P. Reyes, Haiping Hao, Yarira Ortiz-Alvarado, Charles A. Cuff, Eddie Pérez Claudio, Felipe Soto-Adames, Allan H. Smith-Pardo, William G. Meikle, Jay D. Evans, Tugrul Giray, Faten B. Abdelkader, Mike Allsopp, Daniel Ball, Susana B. Morgado, Shalva Barjadze, Adriana Correa-Benitez, Amina Chakir, David R. Báez, Nabor H. M. Chavez, Anne Dalmon, Adrian B. Douglas, Carmen Fraccica, Hermógenes Fernández-Marín, Alberto Galindo-Cardona, Ernesto Guzman-Novoa, Robert Horsburgh, Meral Kence, Joseph Kilonzo, Mert Kükrer, Yves Le Conte, Gaetana Mazzeo, Fernando Mota, Elliud Muli, Devrim Oskay, José A. Ruiz-Martínez, Eugenia Oliveri, Igor Pichkhaia, Abderrahmane Romane, Cesar Guillen Sanchez, Evans Sikombwa, Alberto Satta, Alejandra A. Scannapieco, Brandi Stanford, Victoria Soroker, Rodrigo A. Velarde, Monica Vercelli, Zachary Huang

**Affiliations:** 1 Puerto Rico Science, Technology and Research Trust, San Juan, PR 00927 USA; 2 Present Address: Centre for Life Sciences, Mahindra University, Bahadurpally, Hyderabad 500043 India; 3 Present Address: Florida Department of Agriculture and Consumer Services, Division of Plant Industry, Gainesville, FL 32608 USA; 4 Present Address: Institute of Environment, Florida International University, Miami, FL 33199 USA; 5 Department of Electrical and Computer Engineering, University of Miami, Coral Gables, FL 33146 USA; 6 Office of Institutional Research, Dartmouth College, Hanover, NH 03755 USA; 7 USDA-ARS, Honey Bee Breeding, Genetics and Physiology Research, Baton Rouge, LA 70820 USA; 8 Roy J. Carver Biotechnology Center, University of Illinois, Urbana-Champaign, IL 61801 USA; 9 Data Science and Analytics Innovation Center (dSAIC), University of Missouri-Kansas City, Kansas City, MO 64110 USA; 10 Knight Foundation School of Computing and Information Sciences, Florida International University, Miami, FL 33199 USA; 11 Johns Hopkins University School of Medicine, Baltimore, MD 21205 USA; 12 Department of Biology, University of Puerto Rico, San Juan, PR 00931 USA; 13 Department of Biomedical Informatics, School of Medicine, University of Pittsburgh, Pittsburgh, PA 15206 USA; 14 USDA-APHIS-PPQ, Science and Technology (S&T), Sacramento, CA 95814 USA; 15 USDA-ARS, Carl Hayden Bee Research Center, Tucson, AZ 85719 USA; 16 USDA-ARS, Bee Research Laboratory, Beltsville, MD 20705 USA; 17 University of Carthage, National Agronomic Institute of Tunisia, 1082 Tunis, Tunisia; 18 Honey Bee Research Section, ARC-Plant Protection & Health, P/Bag X5017, Stellenbosch, 7599 South Africa; 19 Forest Fruits Ltd, Lusaka, Zambia; 20 Meltagus, Associação de Apicultores do Parque Natural do Tejo Internacional, 6000-790 Castelo Branco, Portugal; 21 Institute of Zoology, Ilia State University, 3 Giorgi Tsereteli Street, 0162 Tbilisi, Georgia; 22 Facultad de MedicinaVeterinaria y Zootecnia, Departamento de Medicina y Zootecnia de Abejas, Conejos y Organismos Aquáticos (DMZ:ACyOA), Universidad Nacional Autónoma de México, 04510 Ciudad de Mexico, CP Mexico; 23 Applied Chemistry Laboratory, Semlalia Faculty of Sciences, University Cadi Ayyad, Marrakech, Morocco; 24 Amateur Beekeeper, Oviedo, FL 32765 USA; 25 Cochabamba Beekeepers Federation (FEDAC), Aniceto Padilla, 493, Cochabamba, Bolivia; 26 INRAE, French National Research Institute for Agriculture, Food and Environment. UR Abeilles et Environment, 84914 Avignon, France; 27 Institute of Earth Systems, Rural Sciences Farmhouse, University of Malta, Msida, 2080 MSD Malta; 28 Centro de Biodiversidad y Descubrimiento de Drogas, Instituto de Investigaciones Científicas y Servicios de Alta Tecnología (INDICASAT AIP), Clayton Panama, 0843-01103 Panama; 29 Instituto de Ecología Regional (IER), Universidad Nacional de Tucumán (UNT) - Consejo Nacional de Investigaciones Científicas y Técnicas (CONICET). Yerba Buena, CC 34, CP 4107 Tucumán, Argentina; 30 School of Environmental Sciences, University of Guelph, 50 Stone Road East, Guelph, ON N1G 2W1 Canada; 31 Biology Department, Middle East Technical University, 06530 Ankara, Turkey; 32 International Centre of Insect Physiology and Ecology, Nairobi, Kenya; 33 Molecular Biology and Genetics Department, Kilis 7 Aralık University, Kilis, Turkey; 34 Dipartimento di Agricoltura, Alimentazione e Ambiente (Di3A), Università Degli Studi Di Catania, Catania, Italy; 35 Independent Beekeeper, 6000 Castelo Branco, Portugal; 36 South Eastern Kenya University (SEKU), JXFW+X3C, Kitui, Kenya; 37 Department of Agricultural Biotechnology, Tekirdağ Namık Kemal University, 59030 Tekirdağ, Turkey; 38 Professional Training in Livestock and Animal Health, High School Lope de Vega, Fuente Obejuna, Córdoba, Spain; 39 Istituto Zooprofilattico Sperimentale della Sicilia, 90129 Palermo, Italy; 40 Chkhorotsku Local Historical Museum, David Aghmashenebeli St., 5000 Chkhorotsku, Georgia; 41 Escuela de Agronomía, Sede del Atlántico, University of Costa Rica, Turrialba, 30501 Costa Rica; 42 Department of Agricultural Sciences, University of Sassari, Viale Italia 39A, 07100 Sassari, Italy; 43 Instituto de Genética Gv IABIMO, INTA-CONICET, Buenos Aires, Argentina; 44 Agricultural Research Organization, The Volcani Center, Institute of Plant Protection, Department of Entomology, Bet-Dagan, Israel; 45 Bolivian Apiculture Institute (IAB), PROMIEL-SEDEM, Jaimes Freyre No 2344, La Paz, Bolivia; 46 Monica Vercelli Independent Researcher, Turin, Italy; 47 Department of Entomology, MSU Apiculture Lab, Michigan State University, East Lansing, MI 48824 USA

**Keywords:** Honey bee, SNP, Invasive, Diagnostic, Hierarchical agglomerative clustering, Network

## Abstract

**Background:**

Honey bees are the principal commercial pollinators. Along with other arthropods, they are increasingly under threat from anthropogenic factors such as the incursion of invasive honey bee subspecies, pathogens and parasites. Better tools are needed to identify bee subspecies. Genomic data for economic and ecologically important organisms is increasing, but in its basic form its practical application to address ecological problems is limited.

**Results:**

We introduce HBeeID a means to identify honey bees. The tool utilizes a knowledge-based network and diagnostic SNPs identified by discriminant analysis of principle components and hierarchical agglomerative clustering. Tests of HBeeID showed that it identifies African, Americas-Africanized, Asian, and European honey bees with a high degree of certainty even when samples lack the full 272 SNPs of HBeeID. Its prediction capacity decreases with highly admixed samples.

**Conclusion:**

HBeeID is a high-resolution genomic, SNP based tool, that can be used to identify honey bees and screen species that are invasive. Its flexible design allows for future improvements via sample data additions from other localities.

**Supplementary Information:**

The online version contains supplementary material available at 10.1186/s12859-024-05776-9.

## Introduction

Pollinators are critical in maintaining ecosystem functions and serve as primary contributors to the world’s food security [1]. The domesticated western honey bee (HB), *Apis mellifera* Linnaeus 1758, is the premier world pollinator, its contribution to agricultural economies is estimated to be from $200 billion to $351 billion USD/year globally [2–5]*.* The partnership between HBs and humans has a long history [6, 7], and much of their prevalence across the globe is tied to our own spread as a species [8, 9]. It is this history that has made the study of honey bee genetics both interesting and challenging.

The importance of HBs to world food security makes this organism a critical focus of study. This is particularly relevant, because as with many other arthropods, HBs are experiencing seasonal declines [5, 10–12]. The challenges facing HB populations stem from a confluence of management and anthropogenic factors [13–17]. Of these, one is uniquely tied to their association with humans, the potential worldwide spread of novel pests and pathogens. The ready and easy movement of HBs across the world poses a challenge to their health. Movement of HBs across the world also poses a management challenge. Specifically, the uncontrolled introduction of novel genetic variation can be disruptive and negatively affect local apicultural economies. One example is the introduction of *Apis mellifera scutellata* Lepeletier 1836 to the Americas. Beginning from the seventeenth century, and with *A. m. mellifera,* Linnaeus, 1758, honey bees were introduced to the American continent to benefit the honey bee industry [6, 18–22]. In contrast, the accidental release and dispersal of *A. m. scutellata* from a breeding program in Brazil [23] forced changes in existing agricultural practices in the Americas. For instance, in Mexico, presence of Africanized bees resulted in preference for smaller, isolated apiaries and increased number of smaller honey harvests to manage the increased defensiveness of the hives, e.g. [24]. Practice and regulations related to the movement of bees within and across countries also have changed (see [22]). Both health and management challenges have highlighted the need to trace population sources, motivating the development of cost-effective tools to accurately identify the source of HB populations [25–28]. The honey bee was one of the first eukaryotic organisms to have its genome sequenced [29]*.* This resource along with other molecular data published since that time, has permitted the development of methods to track HBs, and their pests. These resources also assist efforts to monitor other pollinators or invasive species [30], for whom genome data may be sparse [31, 32].

Strategies to identify the sources of HB populations have varied. Efforts have capitalized on anatomical markers such as wing venation [33–39], which has been widely adopted and, in some instances, automatized [35, 36, 38, 39]. Genetic approaches have also been implemented, with initial strategies utilizing mitochondrial genes such as cytochrome oxidase I and II [40–42], cytochrome b [43], and ND2 [44], as well as the complete mitochondrial genome [45–47]. Homologous approaches using microsatellites [48, 49], restriction fragment length polymorphisms, RFLPs [44], random amplified polymorphic DNA, RAPDs [50], and microarray-based comparative genomic hybridization, aCGH [51], have also been widely used. More recently, efforts using next-generation sequencing (NGS) technology have become prevalent due to their greater resolution and accuracy [9, 29, 36, 52–61]. The approaches currently in use for population identification of HBs are useful but possess limitations, such as, time required to process the information (e.g., wing venation analysis), or in cost-to-benefit ratio of information (e.g., NGS). Another limit is resolution, for example wing venation patterns can discriminate distantly related species of *Apis* but are not useful at the population level within species. Mitochondrial markers can be used to discriminate major HB lineages (see [62], for review) but cannot readily differentiate subspecies and populations.

Over 20 and up to 33 subspecies of honey bees [62] are divided into four major lineages identified by morphological and molecular data: A (African), O (Near East and Central Asia), M (Western and Northern Europe), and C (Eastern Europe). The African tropical or subtropical origin of HB, *Apis mellifera* is supported by various molecular studies. Bees spread to Europe via two routes, from North Africa via the Iberian and the Arabian Peninsulas and Anatolia. This resulted in a secondary contact between the divergent M and C lineages [52, 53, 57, 63]. Secondary contact also occurred between A and M lineages [64]. In fact, genetic distribution patterns can be better understood by considering secondary contact hypotheses in addition to clinal variation [64]. Currently the natural *A. mellifera* population extends to Central and Southwest Asia, Europe, and Africa. HBs were also introduced to East and Southeast Asia, Australia, and the Americas, by humans [9, 65]. The long history of admixture of HBs due to their association with humans makes it a challenge to accurately discriminate individuals at the population level.

Areas with hybridizing populations pose a particular challenge. In the Americas, the hybridization of *A. m. mellifera* and *A. m. scutellata* has yielded a range of populations with unique genetic variants. In some cases, the genetic variation can be desirable. For example in at least one documented case, *A. m. scutellata* hybridization and local adaptation on the island of Puerto Rico (PR) [66], resulted in a unique combination of reduced defensiveness and mite resistance traits, that enhances its survival [67–69]*.* Other unique HB populations have been documented in the Macaronesia archipelagoes (Azores, Madeira, Canary Islands) [70–74], Balearic Islands [75, 76], Cyprus [75, 76], and Malta [77]. Complex population structure in HB populations has also been observed in places of historical divergence such as differences between mainland African HB populations and those in the Southwest Indian Ocean archipelagos (Mascarene, Seychelles, and Comoros) and Madagascar [78]. The Hawaiian Islands have also reported a unique and locally common haplotype of *A. m. mellifera* [79], although, in this case, selection may have contributed to the emergence of this haplotype. In many of these cases, the ready and cost-effective identification of populations is limited by the lack of resolution of current approaches.

One method that retains resolution while reducing costs is the use of single nucleotide polymorphisms (SNP). Panels of SNPs that are representative of genome-wide variation provide subspecies-level resolution while drastically reducing processing costs [9, 53, 56, 57, 59, 60]. Diagnostic panels have been recently used to monitor the introduction and dispersal of African and Africanized HBs to Australia [56]. Similar strategies have been used to differentiate and track the movement of HBs in other parts of the world, e.g., Eurasia [54], Europe [55, 80–82], Canada [83], and South Africa [36, 58]. These previous studies were restricted to few subspecies of a particular continent or region.

In this work we outline a SNP panel-based approach, HBeeID, which uses information from 272 SNPs and a knowledge-based network analysis to accurately identify HBs at the population level. The tool incorporates a reference set, minimizing the work needed by a user, while also providing greater automation. Using this novel approach, we characterize populations from across the world and use published HB data to test HBeeID’s performance in detecting populations within regions of high admixture and complex population architecture. We posit that this method can become a robust tool for the purpose of identifying and tracking the population source of HBs providing a reliable and cost-effective mechanism to ascertain local and introduced HB genetic variation.

## Implementation

### Newly collected samples

**Fig. 1:**
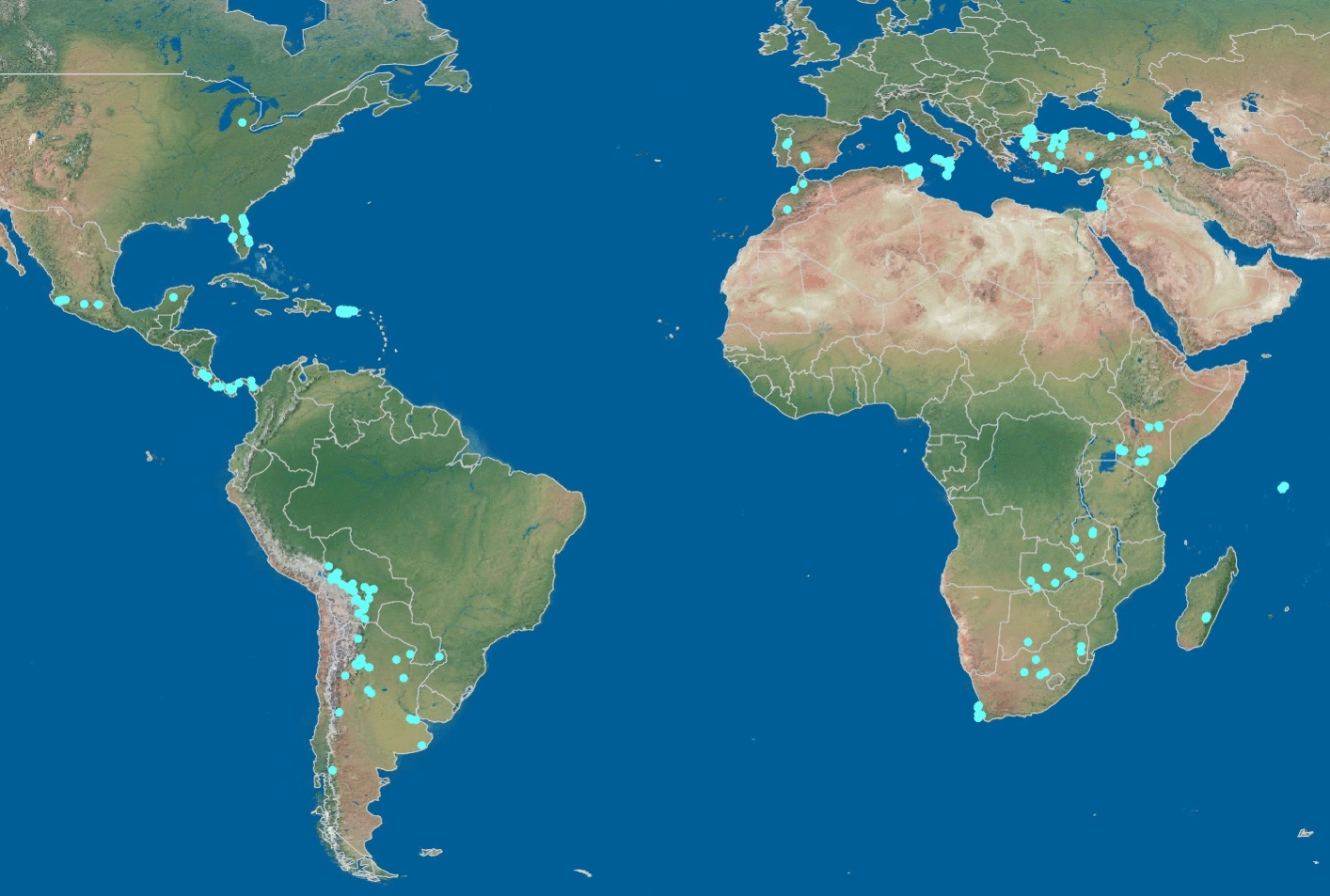
Distribution of honey bee samples. Geographic location of honey bee specimens assayed using the Fluidigm and Agena platforms are indicated by blue dots

**Fig. 2:**
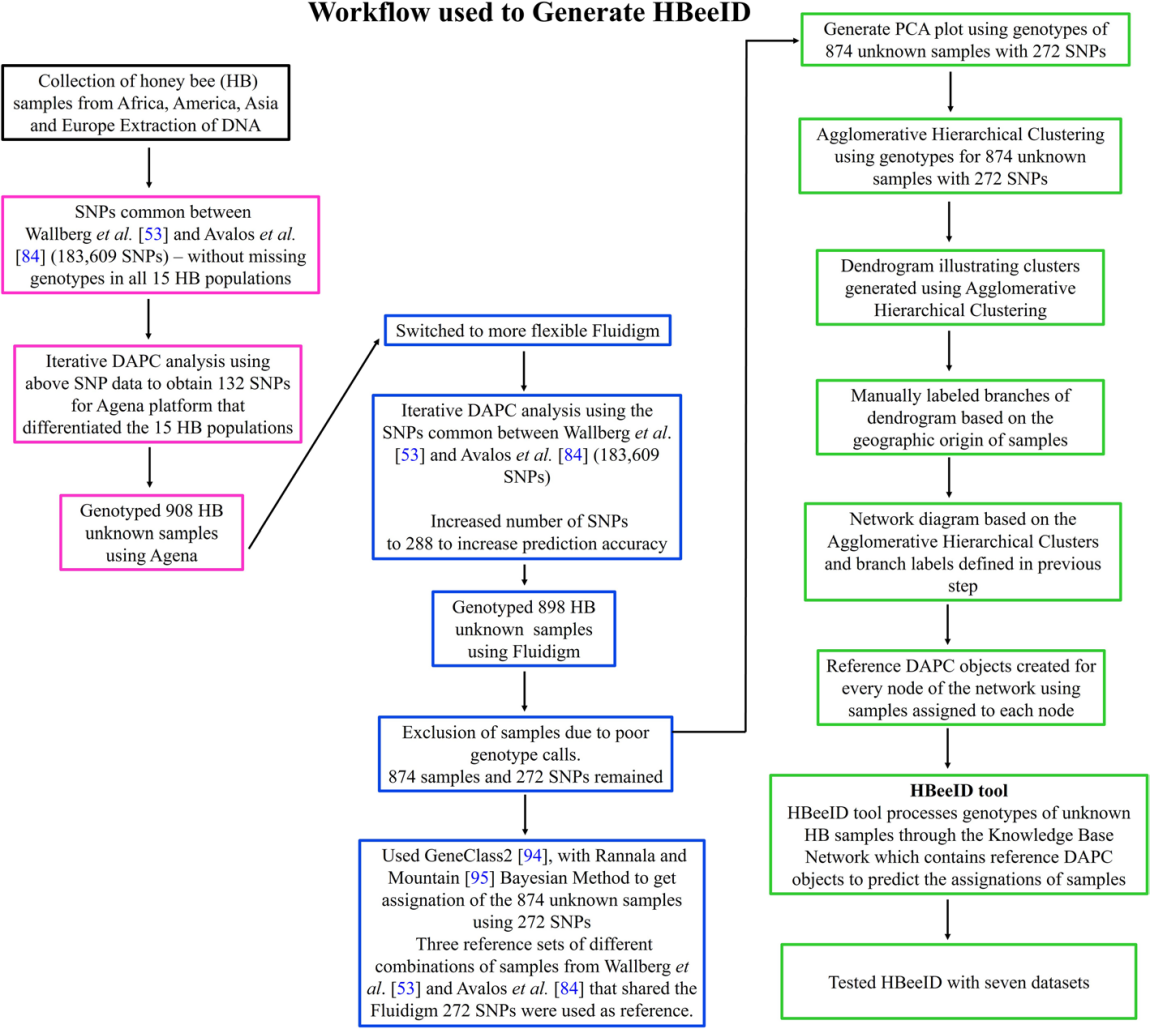
HBeeID tool. Workflow diagram of procedure undertaken to generate the HBeeID tool

The HB samples used for the foundational work to develop HBeeID, henceforth referred to as test HBs, were obtained with the assistance of generous colleagues in the international HB research community. The samples represent a wide geographic area spanning twenty-one countries in Africa, America, Asia, and Europe (Fig. 1). All information related to samples from collector to location (GPS) was recorded digitally and on paper. Sample locality and collector information can be found in (Additional file 1: Tables S1, S2, S3). A workflow diagram of the procedure undertaken to generate the HBeeID tool can be seen in Fig. 2. Details of collection and preservation methods of HB samples can be found in Additional file 2: Methods S1.

### Samples from published data

Using sequence data generated from HBs collected from Puerto Rico (PR), Mexico and Hawaii Avalos et al*.* [84] identified 2,809,085 SNPs. This set of SNPs was combined with published data from Wallberg et al. [53] who identified 8,284,334 SNPs among 12 populations from Europe, Africa, Southwest Asia, and the US. Prior to merging these two data sets, SNP coordinates from the latter were converted to the BeeBase Amel_4.5 genome version, the most recent at the time this work was done. Transformation of the coordinates was done using a mapping file created by aligning SNP flanking sequences against Amel_4.5. As a result of this transformation, it was possible to combine SNPs from the studies of Wallberg et al. [53] and Avalos et al. [84]. The combined dataset consisted of the following populations with the number of samples used written in parenthesis: Puerto Rico (PRHB) (30); Mexico, Africanized HB (AHB) (30); Hawaii, USA, European HB (EHB) (30)﻿ (Avalos et al. [84], *A. m. ligustica,* Spinola 1806 (10), *A. m. carnica,* Pollman 1879 (10), *A. m. anatoliaca,* Maa 1953 (10), *A. m. adansonii,* Latreille 1804 (10), *A. m. capensis,* Eschscholtz 1822 (10), *A. m. iberiensis,* Engel 1999 (10), *A. m. scutellata (10)*, *A. m. syriaca* Skorikov 1929 (10), HB from Sweden (10), Norway (10), Europe (20), and the United States (USA) (20). From the combined dataset a subset of 183,609 SNPs was selected that did not have any missing genotypes in all 15 subspecies or populations.

### Development of the HBeeID tool to predict the assignation of unknown

#### Identification of diagnostic SNPs that differentiate populations

**Fig. 3:**
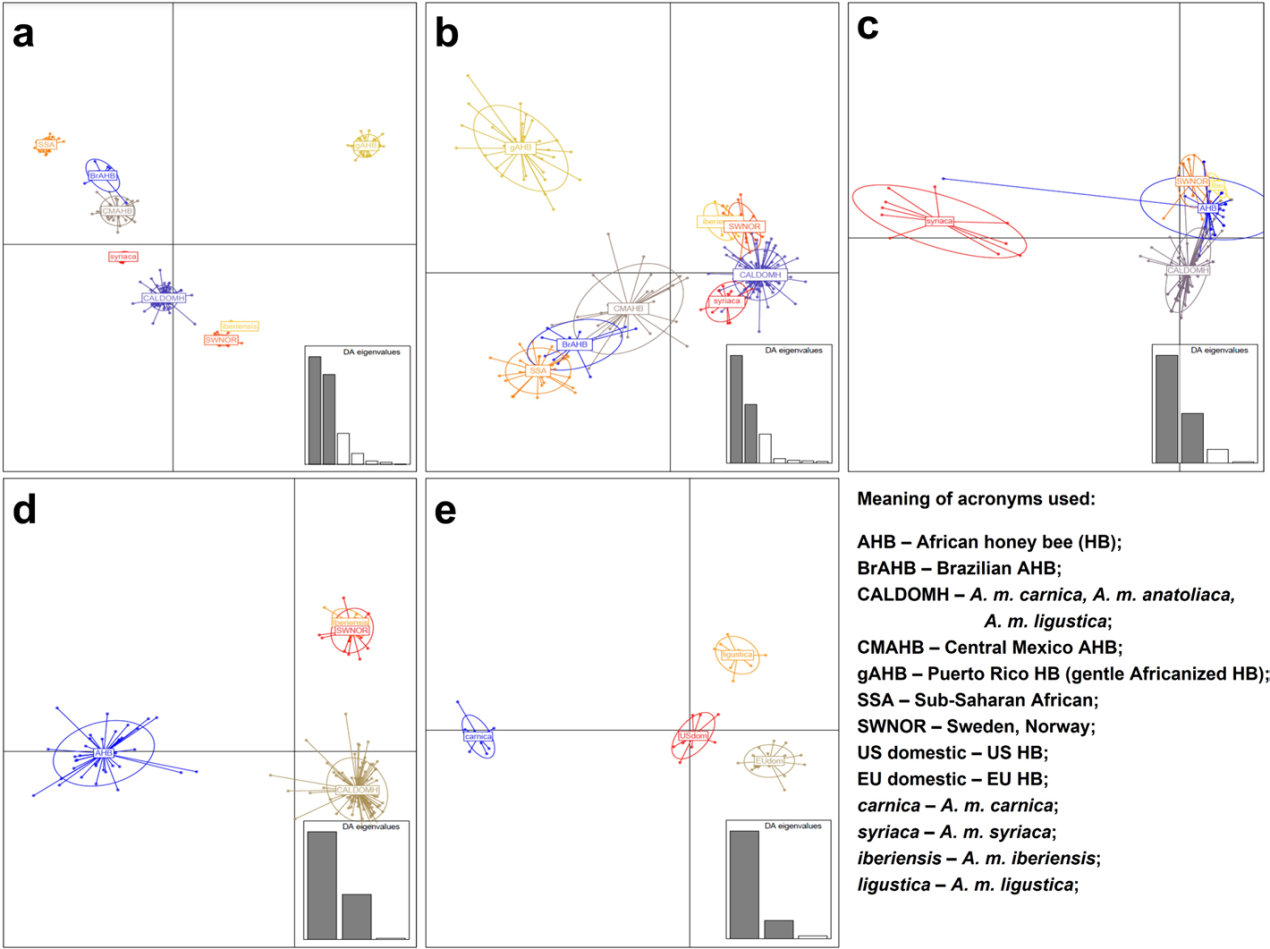
Process to identify diagnostic SNPs. DAPC plots showing clusters of samples of different HB populations used during the process to identify SNPs to differentiate populations. DAPC plots were generated using SNPs that differentiate **a** All samples into eight groups; **b** Puerto Rico HB; **c** *A. m. syriaca* HBs; **d** Africanized HBs; and **e** *A. m. carnica, A. m. ligustica*, EU and US domestic from all other HB populations. Meaning of acronyms used: SSA—Sub Saharan African; gAHB—Puerto Rico Bees (gentle Africanized HB); BrAHB—Brazilian AHB; CMAHB—Central Mexico AHB; US and EU Domestic; SWNOR—Sweden, Norway; US domestic—US HB; EU domestic—EU HB; *carnica*—*carnica* HB; *syriaca*—*syriaca* HB; CALDOMH—*carnica, anatoliaca, ligustica*; *iberiensis*—*iberiensis* HB; *ligustica*—*ligustica* HB

Of the 183,609 common SNPs (See file on github (https://github.com/taoyudong/HBeeID) mentioned in the Implementation section, 7,069 were free of SNPs in the upstream and downstream flanking 32 bases, a requirement to develop good quality oligos for the Agena genotyping assay. These 7,069 SNPs were used to perform several rounds of discriminant analysis of principle components (DAPC) to identify the SNPs that differentiate the 15 populations from the studies of Wallberg et al*.* [53] and Avalos et al*.* [84]. The R package Adegenet [85] was used to cluster individuals with similar SNP genotypes. To determine the minimum number of SNPs that identify a specific population we used an iterative and sequential approach to progress from the broad group categories to the individual subspecies and population level. An initial DAPC run with all SNPs in the combined data set (Fig. 3), generated eight broad clusters that included 15 populations. From this run, SNPs with the highest Linear Discriminant values (LD values) that generated clearly discriminated group clusters were identified. These high-performance SNPs were used in subsequent DAPC runs to determine if at least one of the 15 populations would cluster without overlapping with other groups (Fig. 3). If these smaller sets of SNPs did not separate the test group, DAPC was re-run with additional high LD value SNPs. If the newly added SNPs facilitated the discrimination of the groups, they were retained, and the entire set of SNPs was considered diagnostic for that group. If the newly added SNPs were not found to be useful the process was repeated until SNPs found to be diagnostic for the population in question were identified. This process was repeated until SNPs that differentiated all the populations were determined (Fig. 3).

#### Development of Fluidigm SNP panel

We first tested the Agena system, and due to drawbacks associated with it we changed to the Fluidigm platform, and also increased the number of SNPs from 132 to 288. Moreover, the number of samples from Puerto Rico, one of the critical populations we aimed to discriminate, was also increased. Fluidigm’s high-throughput SNP genotyping platform (Juno Genotyping IFC (Integrated Fluidic Circuits) using SNP Type Assays) incorporates pre-amplification and genotyping on integrated IFC’s. Sample preparation and pre-amplification is done through thermal cycling and IFCs are transferred to the instruments, BioMark or EP1 reader for capturing fluorescent images, these are then analyzed by the Fluidigm Genotyping Analysis software to generate SNP calls. The subset of SNPs, determined to differentiate the 15 HB populations, with 200 SNP-free flanking bases on either side of the SNP loci were sent to the Fluidigm Assay Design Group for the design of SNP primers. SNPs with poor quality primer design scores were removed. A panel of 288 SNPs with high quality primer design scores were retained. Of these 288 SNPs, 16 were excluded from all downstream analysis because these SNPs were found to be homozygous in all samples genotyped. Four samples were found to have more than 50% of genotypes missing and were excluded from the downstream analysis. In addition, 24 samples from France, one sample from Panama and one sample from Turkey were excluded from the analysis due to poor performance. The final assay of 272 SNP was used to analyze 874 samples. Of the 272 SNPs in HBeeID, one SNP could not be mapped to the Amel_HAv3.1. The remaining 271 SNPs were distributed throughout the 16 *A. mellifera* linkage groups (chromosomes). Linkage group 1, the largest of *A. mellifera*, had the highest number of SNPs (40) while the lowest number of SNPs (10) were mapped on linkage groups 13 and 16, two of the smaller chromosomes (Table 1). The average distance between SNPs ranges from 359 Kbp to 1.4 Mbp.

**Table 1:** List and size of chromosomes of *A. mellifera* Amel HAv3.1 assembly and distribution of HBeeID 271 diagnostic SNPs. One SNP was not mapped to any linkage group

LinkageGroup (Chromosome)	RefSeq ID	Size (MB)	#SNPs
1	NC_037638.1	27.75	40
2	NC_037639.1	16.09	13
3	NC_037640.1	13.62	16
4	NC_037641.1	13.4	24
5	NC_037642.1	13.9	16
6	NC_037643.1	17.79	11
7	NC_037644.1	14.2	14
8	NC_037645.1	12.72	18
9	NC_037646.1	12.35	22
10	NC_037647.1	12.36	13
11	NC_037648.1	16.35	21
12	NC_037649.1	11.51	11
13	NC_037650.1	11.28	10
14	NC_037651.1	10.67	12
15	NC_037652.1	9.53	20
16	NC_037653.1	7.24	10

Primer sequences designed by Fluidigm for these SNPs are found in Additional file 3: Table S4, primers for the Agena assay are in Additional file 3: Table S5 and the accompanying methods are in Additional file 2: Methods 1. Genotype data for the 874 samples for the 272 SNPs, along with sample location information, is given in Additional file 4: Tables S6 and S7. Samples were processed using the Fluidigm Biomark HD and SNPtype Genotyping assays according to the manufacturer’s recommended protocols.

Sample processing was as follows: (1) Each sample underwent an initial preamplification using a pool of SNPtype assays set as follows: [2 ul of each SNPtype Assay (STA) and LSP primer were pooled (96 of each), 16 ul of water was added for a total of 400 ul, STA reactions were assembled as follows: 2.5 ul Qiagen 2 × Multiplex PCR master mix, 0.5 ul SNPtype STA primer pool, 0.75 ul Water and 1.25 of Genomic DNA]; (2) Each sample was amplified with 14 cycles of PCR using the following protocol: (95C 15 min; 14 cycles of 95C 15 s, 60C 4 min); (3) 96 well plates were prepared with SNPtype assay mixes followed by 10X assays: [SNPtype Assay mixes: SNPtype Assay (ASP1/ASP2) 3 ul, SNPtype LSP 8 ul, Water 29 ul, for a total of 40 ul]; [10 ×  assays: 2 × Assay Loading Reagent, 2.5, Water 1.5 ul, SNPtype Assay mix 1.0 ul, for a total of 5.0 ul]. (4) The plate of sample mixes was prepared as follows: [Biotium 2 × Fast Probe Master Mix 3.0 ul, SNPtype 20 × sample loading Reagent 0.3 ul, SNPtype Reagent 0.1 ul, ROX 0.036 ul, Water 0.064 ul, DNA (STA amplification) 2.5, or a total of 6 ul]. A 96.96 Dynamic Array IFC was loaded according to the manufacturer's protocol with the 10X assays and sample mixes. A Fluidigm IFC dynamic array was primed and loaded on 96.96 Fluidigm HX Control. Following priming and distribution of all reagents on the IFC, the plate was transferred to the Fluidigm Biomark for amplification and imaging using the Biomark HD SNPtype 96 × 96 V1 protocol. The metadata for all the samples genotyped using the Fluidigm platforms is given in Additional file 1: Table S2.

#### Development of knowledge base network

Given the distribution and representation of samples in our data set, we posit that they can be used to develop a novel, more nuanced, identification tool. To that end, we used a Knowledge Base Network of clusters generated based on a hierarchical agglomerative clustering (HAC) algorithm using geographic sources for our sample set to better characterize HB samples. The HAC is a specific type of clustering algorithm, where each sample is regarded as a cluster at the beginning, and these gradually merge with those that are similar, forming larger clusters. Since HAC starts from the individual samples in the dataset, it is also called a “bottom-up” clustering approach. In this paper, one of the most used HAC algorithms, the Ward method [86], is adopted, and implementation in R, the Agnes function of the cluster package [87] is deployed to analyze the data matrix of the 272 SNP genotypes for the 874 reference HB samples.

**Fig. 4:**
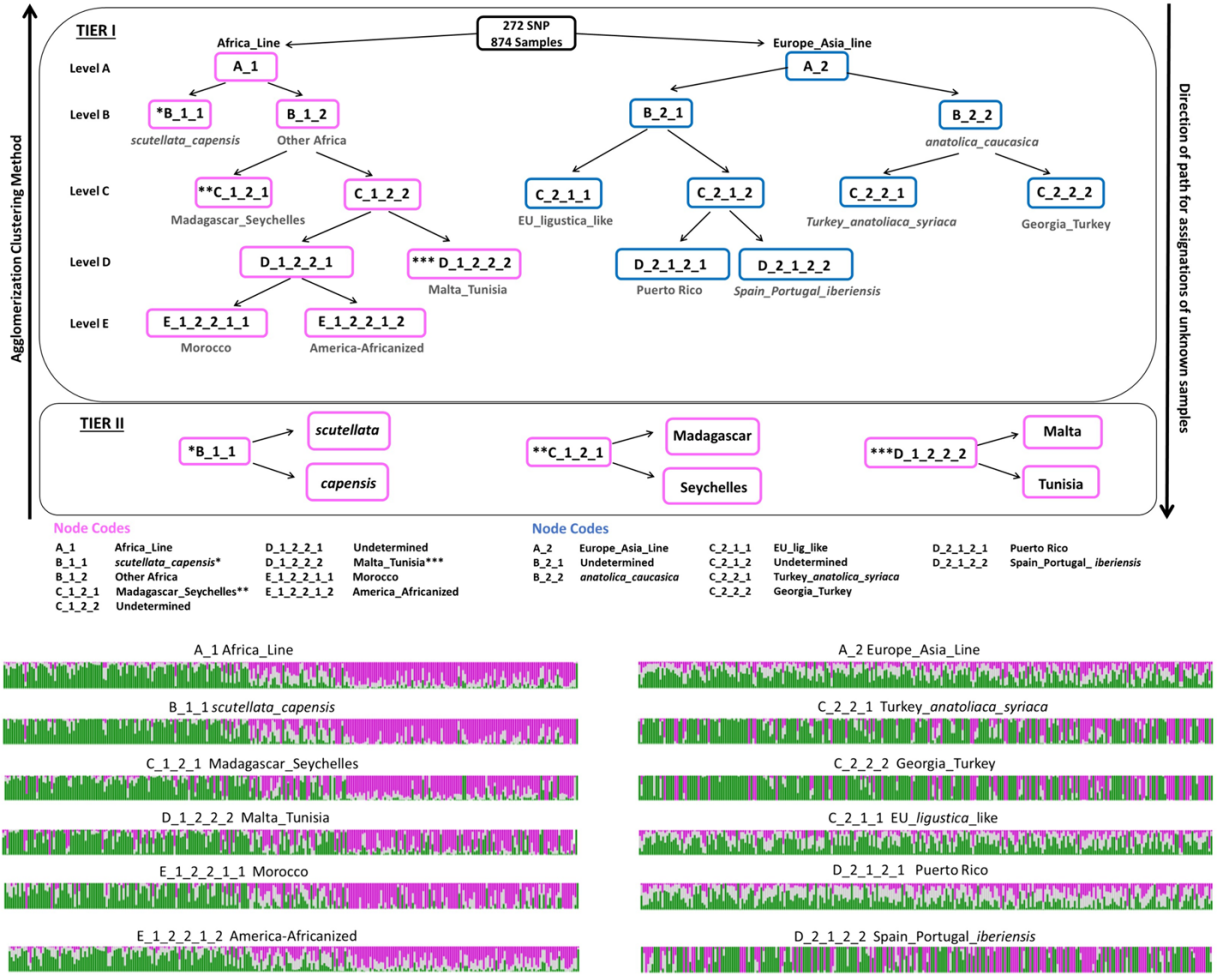
Visualization of hierarchical clustering. Diagram illustrating the agglomerative hierarchical clustering performed, using SNP genotypes, for the development of the Knowledge Base Network used to form HBeeID. Labels indicate the position of the respective groups and the level at which they clustered. The proportional graphs below show the genotype profile of honey bee samples for the 272 SNPs for selected nodes. The length of the bars represents the proportion of 0 (Green), 1 (Gray), or 2 (Pink) genotype in the honey bee samples that comprise each node. The higher the bar the higher the proportion of the corresponding state

Using HAC, the similarity between pairs of samples and the hierarchical structure in the dataset can be easily visualized and interpreted using a dendrogram (Fig. 6). A single sample is the smallest cluster. Related samples will merge to progressively form larger clusters until the single largest cluster point is reached. As the clusters are merged and the number of samples in the cluster increases, the similarities among the samples within a cluster decrease. The height of the placement in the dendrogram reflects the relative similarities among samples. The higher the position of the horizontal line the lower the similarity of the samples, and vice versa. Samples closer to each other are more similar. In the dendrogram, nodes were manually labeled to indicate the geographic locations and/or subspecies of the HB samples present at the branch tips, illustrating the effectiveness of the HAC method for developing the Knowledge Base Network (Fig. 4). Script to generate the proportional graphs in Fig. 4 is provided in Additional file 2: Methods 1.

#### Development of DAPC objects for each node of the dendrogram

The Knowledge Base Network (KBN), whose development is described in the previous section of Implementation, consists of a representation of the relationships between the reference samples at different levels of organization. The reference samples proceed from a general grouping of all samples to specific subgroups of closely related samples. At the origin of the KBN, all the 874 reference samples are present as one cluster with their assignation as belonging to either African or European lines. This assignation was based on results from the hierarchical agglomerative cluster, which used similarities of SNP genotypes to group the reference samples. Groups of samples were then labeled as per their corresponding geographic origin. As an unknown sample proceeds through each node of the KBN, it is compared to each node’s specific grouped reference samples. And, depending on its affinity, it is diverted to the subsequent node whose reference samples it most closely matches. The process is repeated until the unknown sample reaches the end of the network.

To create a tool that allows the assignation of unknown samples, it was necessary to generate a structure that contained genotype information of the respective reference samples that belonged to each node. This structure, which we refer to as HBeeID, consists of a series of R objects generated for each node that include the genotype information of the reference samples as per their respective groups to which they are assigned. Each of the R objects was generated by using the cross-validation function {xvaldapc}, which uses the group assignation of 90% of the samples as the training dataset {training.set = 0.9} and the remaining 10% as test samples.

HBeeID consists of R code reflecting a series of predict-functions which at each node take the appropriate R object, described above, as input along with the SNP genotypes of an unknown sample to be identified. After processing at each node, the unknown sample is then directed to the following nodes to which it has the greatest affinity. The process continues until the final assignment for the unknown sample is reached.

#### Development of the HBeeID tool to predict the assignation of unknown samples

The HBeeID tool presented herein is an R-based tool that utilizes genotype SNP data to determine the assignation of unknown samples by matching the SNP profile of the unknown sample with that of the reference samples. The reference dataset used by HBeeID to predict the assignation of the unknown samples consists of 874 reference samples and their respective genotypes for 272 SNPs. The process of matching the SNP profile of the unknown samples with reference samples takes place at different levels (Tier I—Level A, B, C, D, and E and Tier II), as illustrated in Fig. 4. At the Tier I-level A, Predict Function takes as input the DAPC object containing information as to the genotypes of the 874 reference samples assigned to the A_1 (Africa Line) and A_2 (Europe/Asia Line) nodes along with the 272 SNP genotypes of the unknown samples. The unknown samples are assigned to either the A_1 or A_2 line based on the similarity of SNP genotype profiles. Assignment at subsequent levels (B-E) and nodes proceeds in the same manner. The unknown samples receive their final assignation at the terminal nodes of level D for the Europe/Asia Line, and Level E for the Africa Line. The final assignations for the unknown samples are then exported. Unknown samples that in Tier I are assigned to nodes B_1_1 (*scutellata*/*capensis*); C_1_2_1 (Madagascar/Seychelles), and D_1_2_2_2 (Malta/Tunisia) receive two assignations. To obtain single assignations, the genotypes of these samples along with DAPC objects containing the genotypes of reference samples were given as input to the TIER II level.

### Population genomics

#### Principle component analysis

**Fig. 5:**
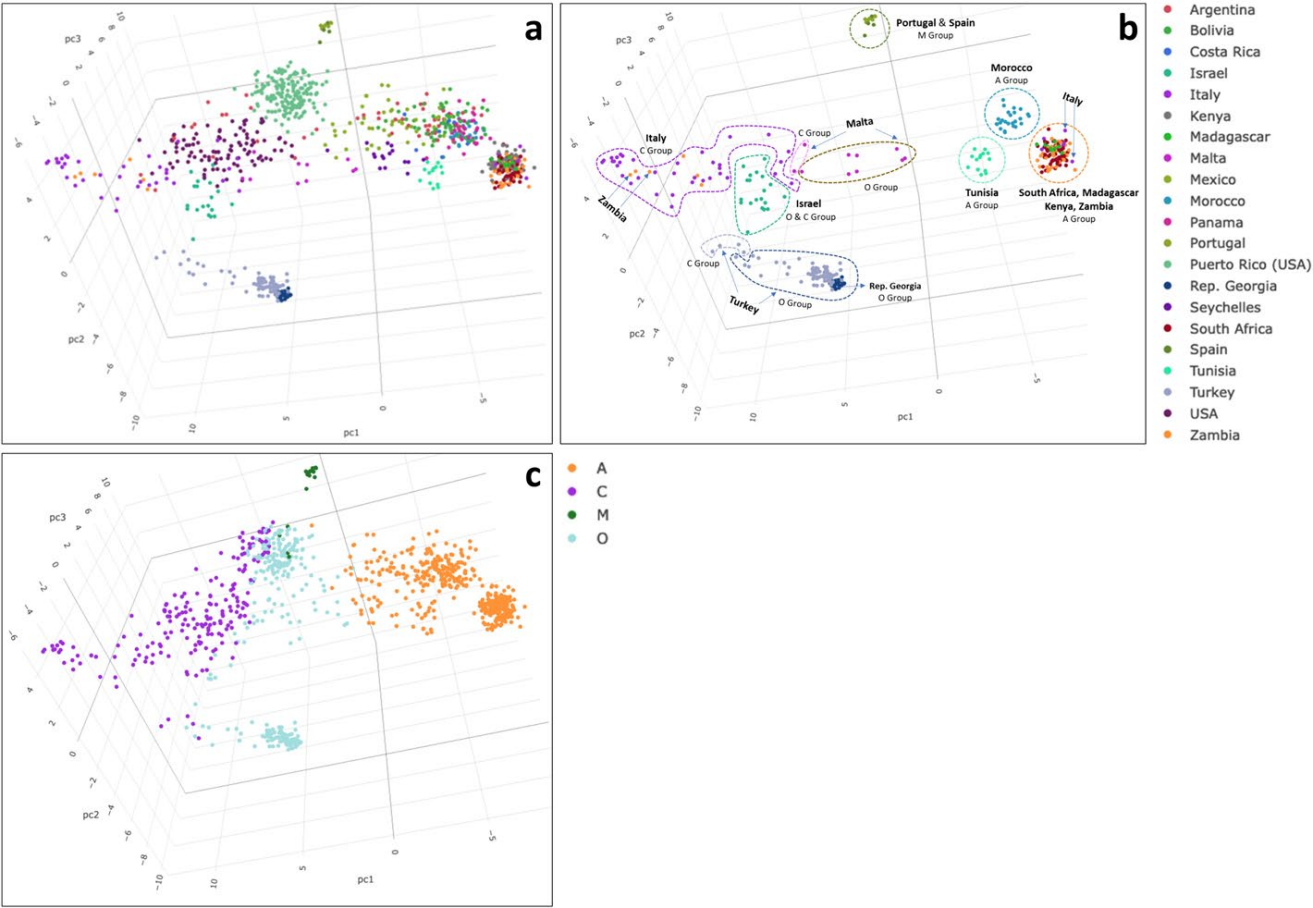
**a**, **b**, **c**. **a** Genotypic relationship of honey bee samples. PCA plot generated using 874 HB samples genotyped with 272 SNPs using the Fluidigm genotyping platform. The reader is encouraged to see the interactive 3D version of this figure available to download in the supplementary section as Additional file 5_3D interactive plot and on github or Additional file 5 (html interface). **b** PCA plot of a subset of all samples from Africa, Italy, Malta, Israel, the Iberian Peninsula, Turkey, and the Republic of Georgia demarcated as per the HB group assignation given when using reference set III. **c** PCA of HB samples identified as per their assignation to the A, C, M, or O groups obtained in the GeneClass2 analysis run with reference set III

Principle Component Analysis (PCA) was used to visualize the segregation of the 874 HB samples utilized to generate HBeeID (JMP, Version 14. SAS Institute Inc., Cary, NC, 1989–2021) (Fig. 5a). The main text includes an image of component 1 of the PCA analysis (Fig. 5a), but we strongly encourage the reader to also see the results of this analysis as an interactive three-dimensional PCA plot in Additional file 5, where the relationship between samples can more accurately be seen in three-dimensional space, using different perspectives, and by selecting samples from different countries.

Prior to the analysis, a singular value decomposition (SVD) imputation was performed on the 272 SNP genotypes across all 874 samples to replace missing genotypes with imputed values. Imputed genotypes for the 272 SNPs for all 874 samples were given as input to the principal component function under the multivariate methods of JMP to generate Principal Component 1 (PC1) and Principal component 2 (PC2), these were imported into the graph builder function to generate PCA plots that visualized the relationship of the reference samples to each other. Principle component analysis is a variable reduction technique that finds a linear combination of variables that explains the variance among the samples. The PCA plots therefore represent the similarities of samples when considering all the SNPs included in the analysis and allow us to plot in only two dimensions.

To further illustrate the genetic variation of collected samples, a subset of all samples from Africa, Italy, Malta, Israel, the Iberian Peninsula, Turkey, and the Republic of Georgia were further demarcated as per the HB group assignation given results from using reference set III (Fig. 5b), in addition HB samples were identified in the PCA as per their HB group assignation of A, C, M, or O (see Impact of reference datasets on assignations) obtained in the GeneClass2 analysis run with reference set III (Fig. 5c).

#### Identification of SNPs from publicly available datasets

To evaluate the performance of the HBeeID, we used sequences of HB samples from published studies as unknowns, and identified SNP’s to be used for the testing.

Sequence data from Harpur et al*.* [52], Cridland et al*.* [57], Harpur et al*.* [88] were downloaded using the NCBI BioProject IDs, (PRJNA216922; PRJNA385500; PRJNA363032, respectively) provided in the manuscripts. Read quality check was performed using FASTQC (http://www.bioinformatics.babraham.ac.uk/projects/fastqc). To trim low quality bases as well as any traces of adapter bases from the sequencing reads trimmomatic [89] software was used with parameters ILLUMINACLIP:TruSeq3-PE-2.fa:2:30:10:5 LEADING:30 TRAILING:30 SLIDINGWINDOW:3:15 MINLEN:30. This ensures trimming of bases with quality score below Q30 from the 5’ and 3’ ends of the reads and also removes the entire 3’ part of the read when the average quality score in a window of 3 bases falls below Q15.

High quality trimmed reads from all the samples were aligned against the HB reference genome, (Amel_HAv3.1) downloaded from NCBI Genome database, using BWA-MEM aligner (version 0.7.17) [90] using -M which marks shorter split hits as secondary and -R that allows to specify read group information, along with all default parameters. Read alignments in SAM format were converted into BAM format using SAMtools (version 1.7) [91]. Unsorted alignments in BAM format were sorted and then indexed using SAMtools. Picard tools (http://picard.sourceforge.net/) function MarkDuplicates was used to tag duplicate reads within the BAM file. To identify raw variants for each sample, alignment files in BAM format that are coordinated sorted and marked with duplicates were base quality score recalibrated using the Genome Analysis Tool Kit (GATK) version 3.8. Recalibrated BAM files were given as input to GATK HaplotypeCaller using parameters –emit_mode gvcf to generate GVCF format output,—phasing 1 to include phasing information in the output, and –ploidy 2 to consider the input sample as diploid. This resulted in the generation of a VCF file for each sample. Individual VCF files from all the samples were given as input to Sentieon [92] using –algo GVCFtyper option to perform joint variant calling which generated a single VCF file with genotype information for all the raw variants in all the samples. To quality filter the variants, variantFiltration program of GATK was run using parameters: QUAL < 30 to retain only variants that could be false positives with a probability of 0.001, QualByDepth(QD) < 2.0, variants below this threshold were empirically determined to fail machine learning based VQSR filtering, RMSMappingQuality (MQ) < 4.0, which indicates the root mean square quality of all the reads at the variant site is very low, MQRankSum < − 12.4, suggests that mapping qualities of the reads carrying reference allele are significantly higher than those reads supporting the alternate allele, ReadPosRankSum < − 8.0, which indicates that alternate allele are mostly identified near the ends of the reads, FisherStrand (FS) > 60.0, which is an indication of a bias between forward and reverse strands for reference and alternate alleles and StrandsOddRatio (SOR) > 3.0, another measure to determine strand bias. Script to convert haploid genotypes in VCF format to phased diplotized genotypes in VCF format is provided in Additional file 2: Methods 1.

#### Testing of HBeeID with published honey bee data sets

To test the performance of HBeeID we used a data set produced in our laboratory and six other published data sets listed in Implementation (Impact of reference datasets on assignations). SNPs for all data sets were extracted using the Amel version HAv3.1 and the genotype data converted to [0, 1 or 2], where [0] represents the homozygote state for the reference allele, (1) represents the heterozygote genotype, and (2) represents the homozygote state for the alternate allele. The file was formatted in the manner suitable for submission to HBeeID. See Additional file 2: Methods 1, for details on how to run the HBeeID workflow and for file formatting details.

All data sets lacked some of the SNPs present in HBeeID and ranged from three in Wallberg et al. [53] to 74 in Kadri et al. [93] (Table 2). Each HB sample had additional missing SNPs that ranged from 3 to 96 (1 to 35%). A cutoff of 96 missing SNPs was used for the samples included in the HBeeID assessment. Their SNP genotype data, extracted and formatted in a way suitable for giving as input to HBeeID can be found in Additional file 6: Table S8.

**Table 2:** Testing of HBeeID. Results from the testing of HBeeID using honey bee sequence data from this work and publicly available data

Data source	Number of SNPs used of 272 in HBeeID and number of missing SNPs	Original HB identification and total number of samples tested	HBeeID Prediction Descriptors	Total number of HB samples tested	Number of HB samples and respective missing SNPs (Used samples with 96 or fewer missing SNPs)	Percentage match
Current work	257 (15 missing)	Puerto Rico-Africanized (34)	Puerto Rico	33	1(58); 1(59); 1(60); 2(61); 1(63); 2(64); 2(65); 1(66); 2(67); 2(68); 1(69); 4(71); 2(72); 2(73); 1(74); 1(75); 3(76); 3(77); 1(82)	97%
			EU_lig_like	1	1(67)	
Cridland et al. [57]^†^	259 (13 missing)	Northern California (26) M and C Lineage	EU_lig_like﻿	25	1(17); 1(20); 1(22); 1(23); 2(28); 1(30); 3(31); 1(33); 1(38); 2(39); 3(41); 2(42); 1(43); 1(46); 1(47); 1(49); 1(71); 1(77); 1(78)	96%
			Puerto Rico	1	1(28)	
		Southern California: A, M and C Lineage (14)	EU_lig_like	10	1(21); 1(23); 1(25); 1(26); 2(30); 1(32); 1(35); 1(71); 1(72)	29%
			America-Africanized	4	1(18); 1(27); 1(40); 1(63)	
		Avalon (Island): M and C Lineage (4)	EU_lig_like	4	1(17); 1(23); 1(29); 1(96)	100%
Avalos et al. [84]	264 (8 missing)	Mexico-Africanized (28)	America-Africanized	25	8(8); 3(9); 1(10); 1(11); 1(13); 1(14); 1(15); 3(16); 1(17); 3(18); 1(19); 1(22)	
	Puerto Rico	1	1(8)		97% *	
	Madagascar/Seychelles	1	1(8)			
	EU_lig_like	1	1(10)			
	US-European (Hawaii) (30)	EU_lig_like	30	19(8); 10(9); 1(13)		100%
		Puerto Rico-Africanized (30)	Puerto Rico	30	18(8); 8(9); 3(10); 1(20)	100%
Kadri et al. [93]	198 (74 missing)	Brazil-Africanized (26)	America-Africanized	22	10(73); 5(74); 2(75); 2(76); 1(77); 1(83); 1(96)	100% *
			*A. m. scutellata*	4	1(74); 1(76); 1(85); 1(89)	
Wallberg et al. [53]	269 (3 missing)	*A. m. adansonii* (10)	*A. m. scutellata*	10	3(3); 6(4); 1(5)	100% *
	Brazil-Africanized (10)	America-Africanized	10	2(3); 6(5); 1(6); 1(7)		100%
	*A. m. anatoliaca* (10)	Turkey	9	4(4); 3(5); 1(7); 1(10)		100%
		Georgia_Turkey	1	1(5)		
	*A.m. mellifera* EU domestic (20)	EU_lig_like	20	9(3); 6(4); 4(5); 1(6)		100%
	*A. m. mellifer*a US domestic (10)	EU_lig_like	10	2(3); 6(4); 2(5)		100%
	*A. m. carnica* (10)	EU_lig_like	10	3(3); 2(4); 4(5); 1(6)		100%**
	*A. m. capensis* (10)	*A. m. capensis*	5	2(3); 3(4)		50% *
		*A. m. scutellata*	5	2(3); 1(4); 2(6)		
	*A. m. iberiensis* (10)	*A. m. iberiensis*	9	4(3); 1(4); 3(5); 1(6)		90%
		EU_lig_like	1	1(4)		
	*A. m. ligustica* (10)	EU_lig_like	10	1(3); 4(4); 4(5); 1(6)		100%
	*A. m. mellifera* Sweden-Norway (20)	*A. m. iberiensis*	19	7(3); 6(4); 3(5); 2(6); 1(7)		95%***
		EU_lig_like	1	1(4)		
	*A. m. scutellata* (10)	*A. m. scutellata*	10	1(3); 5(4); 3(5); 1(6)		100%
Harpur et al. [52]	258 (14 missing)	*A. m. jemenitica* (10)	America-Africanized	9	6(15); 2(16); 1(23)	100%*
		*A. m. scutellata*	1	1(18)		
	*A. m. scutellata* (11)	*A. m. scutellata*	9	1 (14); 2(15); 3(16); 2(17); 1(18)		100%*
		America-Africanized	2	1(14); 1(15)		
	*A. m. carnica* (9)	EU_lig_like	9	4 (14); 5(15)		100%**
	*A. m. mellifera* Poland (5)	EU_lig_like	1	1(18)		20%
		Puerto Rico	3	1(15); 2(16)		
		*A. m. iberiensis*	1	1(25)		
	*A. m. iberiensis* (4)	*A. m. iberiensis*	4	2 (17); 1 (20); 1(24)		100%
Harpur et al. [88]	225 (47 missing)	*A. m. mellifera* Canada (125)	EU_lig_like	124	99(47); 25(48)	99%
			America-Africanized	1	1(48)	

*Current version of HBeeID identifies African or Africanized honey bee subspecies as either *A. m. scutellata, A. m. capensis*, Americas-Africanized or Puerto Rico Gentle honey bee

**Current version of HBeeID identifies European bee subspecies such as *A. m. carnica* and others as EU_lig_like

***Current version of HBeeID does not differentiate the closely related *A. m. iberiensis* and *A. m. mellifera* Sweden-Norway

HBeeID Prediction Descriptors—Descriptors for HB sub populations used in the HBeeID prediction column:

Puerto Rico—Gentle Africanized HB of Puerto Rico

EU_lig_like—Similar to *A. m. ligustica*

Americas-Africanized- European/African hybrids found in Central, North, and South America

Individual honey bees have different proportions of European and African genes

Madagascar/Seychelles—Similar to honey bees genotyped from Madagascar and/or Seychelles

Georgia_Turkey—Similar to honey bees genotyped from the Republic of Georgia and North East Turkey

^†^Southern California specimens from Cridland et al*.* [57] were collected from 1910 to 2014 and have widely different levels of Africanization. Lineages are as follows: A (Africa); C (Eastern Europe); M (western Europe)

#### Impact of reference data sets on assignations

To illustrate the impact of using different data sets on the assignation of honey bees, especially hybrid individuals, unknown samples genotyped using Fluidigm were also analyzed with GeneClass2 [94, 95], a program that requires the input of a reference data set. Samples were genotyped with the 251 SNPs in common with the reference samples from a total of 272 SNPs in the Fluidigm assay. Assignations of the unknown samples are to the closest available in the two reference sets of samples given as input to GeneClass2. Three reference data sets were used in order to show the effect of using different reference taxa combinations on the resulting assignations: Reference Set I: African/Africanized/EHB Hawaii: 30 PRHB from Puerto Rico; 28 AHB from Mexico and 30 EHB from Hawaii, Avalos et al. [84]; 10 AHB from Brazil and ten each of three subspecies from Africa (SSA, Sub Saharan Africa), *A. m. adansonii, A. m. scutellata*, and *A. m. capensis*, Wallberg et al. [53]. Reference Set II: African/Africanized/EHB Hawaii/EHB Europe and US/Asia: The above data plus the remaining samples from Wallberg et al. [53]: *A. m. anatoliaca* (10); *A. m. mellifera* EU Domestic (20); *A. m. mellifera* US Domestic (10); *A. m. carnica (10); A. m. iberiensis* (10), *A. m. ligustica* (10); *A. m. syriaca* (10)*, A. m. mellifera,* Sweden, Norway, Europe (20). Reference Set III: African/European/Asian: Collapsed populations of main groups from Wallberg et al. [53]; Group M: *A. m. mellifera,* Sweden (10), Norway, Europe (10), *A. m. iberiensis* (10); Group C: *A. m. ligustica* (10), *A. m. carnica* (10); Group O: *A. m. anatoliaca* (10), *A. m. syriaca* (10); Group A: *A. m. adansonii* (10)*, A. m. scutellata* (10), and *A. m. capensis* (10). These specific samples were used as references because they include African and America-Africanized samples including the island of Puerto Rico as well as European and Near East HBs. Genotype data from the three sets of reference samples were run separately, along with the genotypes of the unknown worldwide collection of HB test samples as input for GeneClass2.

**Fig. 6:**
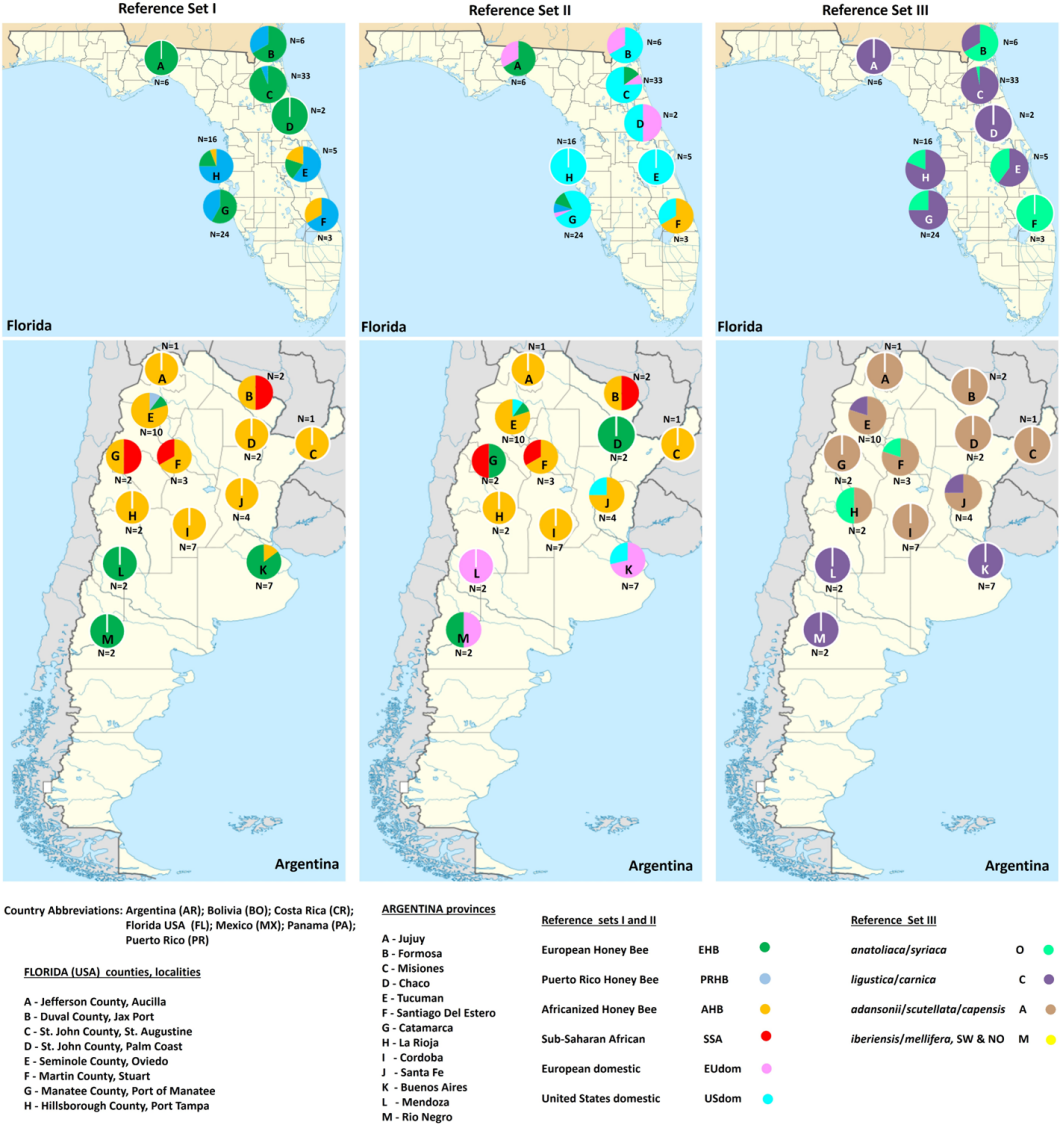
Assignation of honey bees with different reference data. Geographic distribution of samples from Argentina and Florida (US) and their genetic assignations obtained with GeneClass2 using three different combinations of samples from Avalos et al*.* [84] and Wallberg et al*.* [53] as reference

GeneClass2 assigns an individual to a group with the smallest genetic distance [94]. A summary of the GeneClass2 assignation results for all the unknown test samples from the three runs are listed in Table 3. Group categories were assigned following those in Wallberg et al. [53]. The assignations and geographic distribution of samples from Argentina and Florida for reference sets I, II and III are visualized in Fig. 6. The SNP genotypes and individual assignations for all samples run with the three different reference sets can be found in Additional file 7: Table S9. The SNP genotypes in GenePop format for all samples for the three different data sets can be found in Additional files 8, 9, 10, 11: Tables S10–S13.

**Table 3:** Results from the assignation of test samples using the genetic assignment software GeneClass2 (94)

AMERICA									AFRICA								
HB REFERENCE SET I			HB REFERENCE SET II			HB REFERENCE SET III			HB REFERENCE SET I			HB REFERENCE SET II			HB REFERENCE SET III		
Country	N	Sample ID	Country	N	Sample ID	Country	N	Sample ID	Country	N	Sample ID	Country	N	Sample ID	Country	N	Sample ID
**Argentina**	**47**		**Argentina**	**47**		**Argentina**	**47**		**Kenya**	**59**		**Kenya**	**59**		**Kenya**	**59**	
	32	AHB		30	AHB		30	A		59	SSA		59	SSA		59	A
	11	EHB		2	EHB		14	C	**Madagascar**	**14**		**Madagascar**	**14**		**Madagascar**	**14**	
	1	PRHB		0	PRHB		3	O		14	SSA		14	SSA		14	A
	3	SSA		3	SSA				**Morocco**	**25**		**Morocco**	**25**		**Morocco**	**25**	
				8	EU DOM					3	AHB		3	AHB		25	A
				4	US DOM					22	SSA		22	SSA			
**Bolivia**	**60**		**Bolivia**	**60**		**Bolivia**	**60**		**Seychelles**	**16**		**Seychelles**	**16**		**Seychelles**	**16**	
	53	AHB		53	AHB		60	A		16	AHB		16	AHB		15	A
	7	SSA		7	SSA											1	O
**Costa Rica**	**10**		**Costa Rica**	**10**		**Costa Rica**	**10**		**South Africa**	**52**		**South Africa**	**52**		**South Africa**	**52**	
	10	AHB		10	AHB		10	A		52	SSA		52	SSA		52	A
**Mexico**	**46**		**Mexico**	**46**		**Mexico**	**46**		**Tunisia**	**15**		**Tunisia**	**15**		**Tunisia**	**15**	
	46	AHB		46	AHB		38	A		13	AHB		13	AHB		15	A
							8	O		2	SSA		2	SSA			
**Panama**	**36**		**Panama**	**36**		**Panama**	**36**		**Zambia**	**43**		**Zambia**	**43**		**Zambia**	**43**	
	26	AHB		26	AHB		36	A		37	SSA		37	SSA		37	A
	10	SSA		10	SSA					6	EHB		5	EHB		6	O
**Puerto Rico, USA**	**169**		**Puerto Rico, USA**	**169**		**Puerto Rico, USA**	**169**						1	*carnica*			
	165	PRHB		164	PRHB		133	O									
	4	AHB		4	AHB		32	C									
				1	US DOM		4	M									
**USA**	**99**		**USA**	**99**		**USA**	**99**										
	4	AHB		2	AHB		82	C									
	65	EHB		14	EHB		17	O									
	30	PRHB		2	PRHB												
				11	EU DOM												
				70	US DOM												

EUROPE									ASIA								
HB REFERENCE SET I			HB REFERENCE SET II			HB REFERENCE SET III			HB REFERENCE SET I			HB REFERENCE SET II			HB REFERENCE SET III		
Country	N	Sample ID	Country	N	Sample ID	Country	N	Sample ID	Country	N	Sample ID	Country	N	Sample ID	Country	N	Sample ID
**Italy**	**42**		**Italy**	**42**		**Italy**	**42**		**Israel**	**20**		**Israel**	**20**		**Israel**	**20**	
	34	EHB		23	EHB		6	A		20	EHB		8	EHB		13	C
	1	PRHB		0	PRHB		36	C					2	EU DOM		7	O
	1	AHB		0	AHB								10	US DOM			
	6	SSA		6	SSA				**Rep. Georgia**	**26**		**Rep. Georgia**	**26**		**Rep. Georgia**	**26**	
				1	EU DOM					26	EHB		26	*anatoliaca*		26	O
				12	US DOM				**Turkey**	**73**		**Turkey**	**73**		**Turkey**	**73**	
**Malta**	**10**		**Malta**	**10**		**Malta**	**10**			73	EHB		64	*anatoliaca*		68	O
	7	AHB		4	AHB		8	O					8	EHB		5	C
	1	EHB		6	US DOM		2	C					1	US DOM			
	2	PRHB															
**Portugal**	**8**		**Portugal**	**8**		**Portugal**	**8**										
	8	AHB		8	*iberiensis*		8	M									
**Spain**	**4**		**Spain**	**4**		**Spain**	**4**										
	4	AHB		4	*iberiensis*		4	M									

Samples were genotyped with the 251 SNPs in common with the reference samples from a total 272 SNPs in the Fluidigm assay. Assignations of the unknown samples are to the closest available in the two reference sets of samples given as input to GeneClass2. Reference Set I. Avalos et al*. *[84] (30 PRHB from Puerto Rico; 28 AHB from Mexico and 30 EHB from Hawaii); Wallberg et al*.* [53] (10 AHB from Brazil and 10 each of three species from Africa (SSA, Sub Saharan Africa), *A. m. adansonii, A. m. scutellata*, and *A. m. capensis*). Reference Set II. The above data plus the remaining samples from Wallberg et al*.* [53]; *A. m. anatoliaca* (10); *A. m. mellifera* EU Domestic (20); *A. m. mellifera* US Domestic (10); *A. m. carnica (10); A. m. iberiensis* (10), *A. m. ligustica* (10); *A. m. syriaca* (10)*, A. m. mellifera,* Sweden, Norway, Europe (20). Reference Set III. Collapsed populations of main groups from Wallberg et al. [53]; Group M: *A. m. mellifera,* Sweden (10), Norway, Europe (10), *A. m. iberiensis* (10); Group C: *A. m. ligustica* (10), *A. m. carnica* (10); Group O: *A. m. anatoliaca* (10), *A. m. syriaca* (10); Group A: *A. m. adansonii* (10)*, A. m. scutellata* (10), and *A. m. capensis* (10). Genotype data from the three sets of reference samples were run separately along with the genotypes of the unknown worldwide collection of HB test samples as input for GeneClass2. Group categories follow Wallberg et al. [53]

*AHB* African honey bee, *EHB* European honey bee, *PRHB* Puerto Rico honey bee, *SSA* Sub-Saharan African honey bee, *US DOM* United States domestic honey bee, *EU DOM* European domestic honey bee

### Published datasets and how they were used in this study

SNP genotype data obtained from Wallberg et al. [53] and Avalos et al*.* [84] were used in the iterative DAPC analysis  (see previous section) to identify diagnostic SNPs that differentiate HB populations. Data from HB samples generated for this current work; along with those from Wallberg et al. [53]; Cridland et al. [57]; Harpur et al. [52]; Avalos et al. [84]; Kadri et al. [93]; and Harpur et al. [88] with SNPs that overlapped with the 272 SNPs that form HBeeID, were used to test the performance of this new tool.

## Results and discussion

### Population genomics

#### Visualization of genetic relationship of sampled honey bees

The genotype data of the 874 HB samples generated with 272 SNPs was visualized using Principal Component Analysis (PCA). The PCA in Fig. 5a, shows that the HB samples, with few exceptions outlined below, segregate based on their geographic origin. The reader is encouraged to view the same information in the interactive three-dimensional PCA plot in Additional file 5, where the relationship between samples can be seen more readily in three-dimensional space, using different perspectives, and by selecting samples from different countries.

A bird’s eye view of the distribution of samples in the PCA shows them to be in the rough shape of an arrowhead, with Italian samples (from Sardinia) at the very tip to the left, those from the Republic of Georgia (Samegrelo-Zemo Svaneti region) and some Turkey samples (Thrace and Black Sea regions) at the bottom left and African HBs from South Africa, Kenya, Madagascar, and Zambia (with six exceptions) in a tight cluster at the bottom right side of the base of the arrowhead. The countries whose samples form discreet clusters are South Africa, Madagascar, Kenya, Morocco, Tunisia, Republic of Georgia, Portugal, Spain, and Panama. The samples of the remaining countries occur in varied levels of diffused state.

Most samples from a given geographic location are in relative proximity with some notable exceptions. Four samples from Sicily and two from Sardinia, Italy are found at the bottom left along with the African HBs while six samples from Zambia are in the diffuse zone of Italian samples.

Departing from the tight African cluster, at the bottom left, and moving towards the European-like samples at the center of the arrowhead are samples with diminishing levels of African genetic composition. The first samples positioned along this path are samples from Morocco and Tunisia as well as Africanized samples from the American continent, namely, Costa Rica, Panama, Bolivia, Mexico, and Argentina. The samples from the latter two countries have a higher diversity of Africanization levels demonstrated by their long trailing pattern from the African cluster towards the nucleus of samples from Puerto Rico at the center-right of the arrowhead. Samples from Argentina are also found in the center of the arrowhead together with samples from the US, Italy, and other *ligustica-*like samples.

In proximity to the largely America-Africanized cluster are samples from Tunisia and the Seychelles. At the end of the African-Africanized trail, straddling the region between the America-Africanized and the US and other European-like samples, we find the large cluster of 169 samples from the Island of Puerto Rico (PR). This locally adapted island population has been well documented as being of gentle demeanor [67, 84]. None of the samples from Puerto Rico are found in the trail of Africanized samples departing from the African cluster or within it. A reflection of the higher European genetic component of this unique island population.

At the center of the arrowhead, we find the 99 samples from the USA, of which 95 are from Florida and four from Michigan but originally of Georgia stock. These are joined by a subset of samples from southern Argentina, an area documented as a hybrid zone between European and Africanized HBs [96]. In addition, in the center of the arrowhead, are some of the samples from Italy. At the bottom edge of this center group begins the diffused group of samples from Malta, which stretch all the way to the end of the America-Africanized trail of samples and in proximity to samples from the Seychelles and Mexico. The high genetic diversity of the Maltese samples is not surprising for an island that has historically served as a crossroad between Europe and north Africa.

The samples from the Seychelles form a diffused cluster at the end of the trail of the America-Africanized samples. The distinction of the Seychelles HBs from other African HBs, concurs with the findings that HBs from this archipelago form a separate African A1 sub-lineage [78]. The Seychelles group is in the vicinity of the America-Africanized samples as well as samples from Tunisia and three of the samples from Malta. Despite their geographic proximity to the Seychelles, the Madagascar samples group within the African cluster of HBs at the extreme right of the arrowhead. These results also concur with the assignations of SSA for Madagascar HBs and AHB and groups A and O, for HBs from the Seychelles. A difference that is likely the result of human introductions to the latter, as the Seychelles is an island archipelago that has been part of ancient trade routes along the eastern African coastline.

The samples from the Republic of Georgia and some of the samples from Turkey form a tight cluster at the bottom left corner of the arrowhead. The remaining Turkish samples form a stream that orients towards the *A. m. ligustica*-like samples in the center. The Turkish region of Anatolia has served as a region of biodiversity and a bridge between Africa, Europe and Asia, and has been documented to be the home of four subspecies, i.e., *A. m. caucasica* Pollmann 1889*, A. m. syriaca,* and *A. m. meda* Skorikov 1929. An additional fifth subspecies, *A. m. carnica*, occurs in the Thrace region [49, 97, 98].

The samples from Israel form a diffuse group that straddle a region to the right of the *ligustica*-like samples at the center and the end of the stream of samples from Turkey where samples from Thrace and Marmara are located. The native population of HBs in Israel was identified as *A. m. syriaca* [65]*.* The samples we tested indicate that there remains a Southwestern Asian influence in these populations, exemplified by their proximity to Turkish samples, while also sharing genetic similarity to the European-like samples. During the 20th Century, with the development of modern beekeeping in Israel, the original *A. m. syriaca* population was largely replaced with *A. m. ligustica*. The latter is currently actively bred in Israel. However, queens of *A. m. caucasica*, *A. m. carnica* and Buckfast have also been introduced. It is also believed that the wild *A. m. syriaca* population became extinct following the introduction of *Varroa* sp. [99]. Our results concur with those of Henriques et al. [100], who identified samples from Israel as belonging to the C-Lineage.

The European-like samples at the center of the arrowhead are composed of samples from Italy, US, Argentina as well as six of the 44 samples from Zambia, indicating the presence of European HBs in this latter area. As commented earlier, the HB samples from Argentina are very diverse, and they can be found distributed from the African cluster and trailing along the America-Africanized path all the way to the European-like cluster at the center. This distribution reflects the cline that has been documented from the north Brazil-Argentinian border, with more Africanized populations, to the genetically European influenced populations to the south [96].

The endemic HB samples from Portugal and Spain, classified as *A. m. iberiensis* [59, 60, 65], form their own unique cluster, separate and distant from all other samples, at the extreme right, beyond the samples from Puerto Rico.

The PCA analysis of the 874 HB test samples identified discreet populations, confirmed the finding of others and the existence of a high degree of admixture in populations of HBs worldwide. It is difficult to determine categories for the demarcation of admixed HB populations especially when the ancestry of a population is unknown.

#### Impact of reference datasets on assignation

A means by which the identity of an unknown HB test sample can be determined is by comparison to a set of reference samples. To illustrate the effect of using different reference data sets on honey bee annotation, we tested the ability of the 272-SNP Fluidigm-based assay to discriminate the 874 test HBs using the GeneClass2 software with three different combinations of reference samples from published data [53, 84] (see Implementation sections). The GeneClass2 software uses the Monte Carlo resampling algorithm to determine the probability of a sample belonging to a specific reference population [94]. We tested how the assignments of samples changed based on the reference populations used. The composition of the three reference data sets are outlined in Implementation (Testing of HBeeID with published honey bee datasets).

A summary of the assignations obtained using the three data sets is shown in Table 3, and for Argentina and Florida where multiple localities were sampled results are visualized in Fig. 6.

An overview of these results shows that countries whose samples formed very discreet groups in the PCA analysis such as the Sub-Saharan samples (i.e. Kenya, Madagascar, South Africa) were similarly assigned with all three reference data sets. In like manner, samples from Spain and Portugal, with the exception of reference set 1 which did not include *A. m. iberiensis* samples as reference, were consistently assigned as *A. m. iberiensis*. The Africanized samples from Bolivia, Costa Rica, and Panama were also consistently assigned as Africanized or African with all three reference sets. The samples from the Republic of Georgia and most Turkish samples were assigned as *A. m. anatoliaca* when these were included in reference set two and three. The geographic distribution and assignations of the Florida and Argentina samples in Fig. 6 show a southerly distribution of Africanized samples in Florida and northern distribution in Argentina departing from its border with Brazil.

The samples that showed the most marked change in assignation depending on the presence of different European HB reference samples are those from locations that harbor hybrid European-like populations such as Argentina, Italy, Israel, Florida, Puerto Rico and Seychelles illustrating the high degree of admixture in these populations. The ambiguity of these results demonstrate that reference-based discriminations can be limited by availability of data and the accuracy of the classification of individual reference samples. The assignations of HBs from the American continent designated as European, are particularly inscrutable given the lack of precise knowledge of the provenance of the American continent HB-derived reference samples. We know that at least nine HB subspecies may have been introduced to the US since 1622 [21]. In contrast, little is known about specific populations/subspecies introduced to Central and South America outside of *A. m. scutellata* to Brazil [23]. European HB species were likely introduced to the American continent since the colonial period (1492–1810). The earliest records are of introductions in the 1600 s to the Caribbean islands of Barbados and Bermuda [6].

Our PCA analysis confirms prior findings that there is a high degree of admixture in populations of HBs in the American continent and worldwide. An attempt to use the GeneClass2 assignations or PCA analysis to demarcate genetically similar groups would be largely arbitrary and unsatisfactory. An illustration of the difficulty inherent in such an attempt can be seen in Fig. 5b where, for ease of illustration, samples from only a subset of countries have been demarcated as per the assignations obtained with GeneClass2 using the reference set III groupings A, C, M, O from Wallberg et al. [53] in Implementation (Impact of reference datatsets on assignations). Moreover, the classification of all the HB samples tested using these same four groups can be seen in Fig. 5c, which illustrates a continuous stream pattern of diversity in the world populations that we sampled and successfully captured with the 272 SNPs in our assay.

### HBeeID: development, performance, and evaluation

#### HBeeID development strategy

**Fig. 7:**
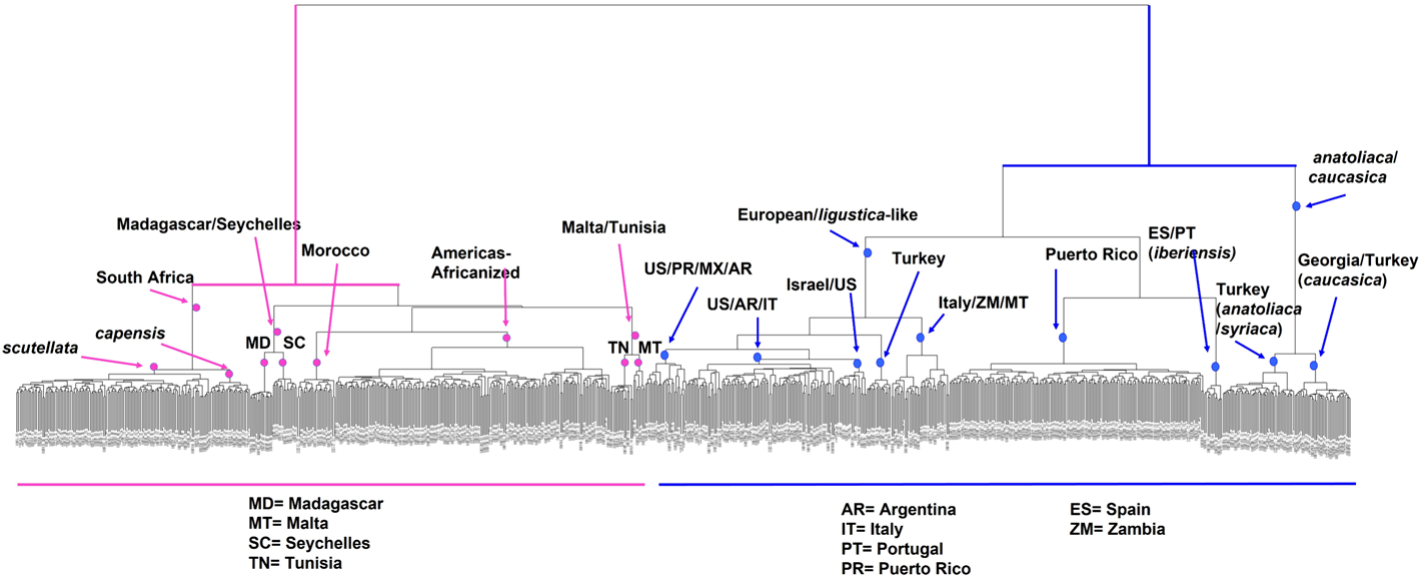
Visualization of agglomerative clusters. Dendrogram that visualizes the agglomerative clusters generated using the Ward clustering method for 874 samples genotyped for the 272 selected SNPs in HBeeID. The countries and their respective abbreviations are listed below the dendrogram

To develop an effective tool to discriminate unknown HBs, it is important to possess reference samples that encompass, as closely as possible, the total genetic variation among the groups one intends to differentiate. However, such attempts are tempered by budget limitations. Our funding made it possible to collect and genotype 874 HB samples from 20 different countries. This work tested the efficacy of the number of samples, the method to identify diagnostic SNPs and the assay type we used to identify unknown HBs. To avoid possible arbitrary determinations of groups, we chose to cluster the 874 HB samples based on similarity of genotypes. Patterns of similarities between HB samples are derived from both geographic proximity and ancestral relationships. Therefore, they can be best described as hierarchical structures. We therefore elected to use the Hierarchical Agglomerative Clustering (HAC) method, which pairs samples based on their similarity, to assign HB samples of similar genotype profiles to different branches of a dendrogram. This method facilitates the delineation of samples into groups and avoids their arbitrary determination (Fig. 7). The HAC method was then used to develop the Knowledge Base Network (KBN) to predict unknown HB samples (Fig. 4). The dendrogram that visualized the relationship of the 874 HB reference samples when using their respective 272 SNP genotypes and the HAC method can be seen in Fig. 7. The organization and relationship of the 874 HB reference samples, based on hierarchical agglomerative clustering, closely resembles the distribution and relationship of these same HBs samples and data when visualized using PCA.

The 874 HB reference samples, their respective 272 SNP genotypes, along with the KBN, an R-based script that utilizes a knowledge base of clusters, form the HBeeID identification tool. HBeeID can make population assignations of unknown HB samples using genotypes based on the 272 selected SNPs. Instructions on how to prepare, format files, and run HBeeID can be found in Additional file 2: Methods 1. Data used to identify diagnostic SNPs for HBeeID are at Github (https://github.com/taoyudong/HBeeID).

#### Testing the performance of HBeeID

The performance of HBeeID was tested using data from this current work, as well as that from six published research studies from Wallberg et al. [53]; Cridland et al. [57]; Harpur et al. [52]; Avalos et al. [84]; Kadri et al. [93]; and Harpur et al. [88]. Results from the tests can be found in: Additional file 12: Table S14 and a summary of the tests for all the data sets can be found in Table 2.

Data from the 34 samples from this current work had 257 (94%) of the 272 SNPs that constitute the HBeeID SNP panel, but other samples had additional missing SNPs that ranged from 58 to 82 of the total number of possible SNPs (21% to 30%) (Table 2). Even with this reduced number of SNPs, HBeeID correctly predicted that 33 of the 34 samples originated from Puerto Rico. One sample with 67 (25%) missing SNPs was assigned to the European *ligustica*-like (EU_lig_like). The assignation of unknown samples by HBeeID is influenced by the differing levels of European and African genome components of a sample, and the number of missing individual SNPs and their specific combination, as these differ in their weight to differentiate HB populations.

The 70 HB samples from Cridland et al. [57] represent HBs from California (CA) with European and/or Africanized identifications and had 259 SNPs (95%) of the 272 HBeeID SNPs. Twenty-six of the 70 samples from this dataset were excluded from the analysis because they had more than 96 missing SNPs. The remaining 44 samples had a range of 17–96 (6–35%) missing SNPs. Of these, 26 were collected from northern CA, 14 from southern CA, and four from Avalon Island, CA. Of the 26 northern CA samples, one was given the incorrect assignation of PRHB while the remaining were assigned correctly as EU_lig_like. Of the 14 samples from southern California, with widely different levels of Africanization (see Cridland et al.)[57]*,* those from Avalon were correctly identified as EU_lig_like (Table 2). To generate assignations Cridland et al. [57] used 3,890,276 SNPs. To evaluate these same samples HBeeID was limited to at most 259 and as few as 176 SNPs. The 272 SNPs in HBeeID, while being distributed on all 16 of the HB chromosomes, cover a mere fraction of the genomic region of the 3,890,276 SNPs used by Cridland et al. [57]. Moreover, if for a given sample the genomic regions represented by the SNPs in HBeeID are not Africanized in a specific admixed individual, HBeeID’s capacity to ascertain whether the sample is Africanized will be reduced.

Of the 88 individuals in the data set from Avalos et al*.* [84], 264 SNPs (97%) were present of the 272 in HBeeID. The number of missing genotypes for these samples ranged from 8 to 22 (3% to 8%). Of the 28 samples identified initially as Mexican-Africanized, HBeeID identified 25 samples as America-Africanized, one as Puerto Rico, one as Madagascar/Seychelles, and one as EU_lig_like resulting in 97% accuracy in predicting the origin of these samples. This data set included an additional 60 samples, 30 labeled as US-European and 30 as Puerto Rico. For both set of samples, the total number of missing genotypes ranged from 8 to 20 (3% to 7%). HBeeID predicted the identity of these samples with 100% accuracy (Table 2).

The 30 samples from Kadri et al. [93] were derived from a pool of 360 individual HBs with an original identification of Brazil-Africanized. These 30 samples had 198 SNPs (73%) of the 272 in HBeeID. Of the different data sets tested, this had the lowest number of SNP genotypes that overlapped with those in the HBeeID SNP panel. Four of the samples were eliminated due to having more than 96 missing SNP genotypes, leaving 26 samples for analysis. Of these 26 remaining samples, 22, with missing genotypes that ranged from 73 to 96 (27% to 35%), were predicted by HBeeID as America-Africanized and four samples, with missing genotypes ranging from 74 to 89 (27% to 32%), were identified as *A. m. scutellata* (Table 2).

The assignation of African HBs that could be built into HBeeID was limited by the genomic data resources available in the public domain at the time this work was conducted and the level of funding to generate the data herein. Hence, it was only possible to develop the capability to identify an African or Africanized HB as either *A. m. scutellata*, *A. m. capensis*, America-Africanized or Puerto Rico-Africanized. As such, the samples from Kadri et al. [93] were identified with 100% accuracy (Table 2).

The 131 specimens from Wallberg et al. [53] include ten HB subspecies and Africanized HBs from Brazil. These samples had 269 SNPs (99%) of the 272 in HBeeID and the least number of missing genotypes, ranging from three to ten (1% to 4%). Given the current limited available reference sequence data resources for closely related European subspecies such as *A. m. carnica* and *A. m. ligustica,* HBeeID identifies these two subspecies as EU_lig_like. With this limitation, HBeeID assigned, with 100% accuracy, samples of *A. m. ligustica*, *A. m. carnica*, *A. m. mellifera* EU domestic samples, and *A. m. mellifera* US domestic from Wallberg et al. [53] as EU_lig_like (Table 2). Of the ten *A. m. iberiensis* samples, nine were identified as *A. m. iberiensis* and one as EU_lig_like, a 90% accuracy. Of the 20 *A. m. mellifera* samples from Sweden and Norway, 19 were identified as *A. m. iberiensis* and one as EU_lig_like. Wallberg et al. [53] determined these samples from Norway and Sweden to be part of the M lineage together with *A. m. iberiensis*, thus the prediction accuracy of HBeeID for these samples is 95%. The 11 *A. m. anatoliaca* samples were identified with 100% accuracy as being samples originating from Turkey or the Republic of Georgia. HBeeID identified the samples of Brazilian-Africanized origin as America-Africanized and the *A. m. scutellata* from South Africa as *A. m. scutellata* with 100% accuracy*.* The ten samples of *A. m. adansonii* were assigned as *A. m. scutellata.* For African HBs, HBeeID is designed to give the assignation of *A. m. scutellata*/*A. m. capensis* in Tier I, and when samples proceed to Tier II, they are differentiated between *A. m. scutellata* and *A. m. capensis.* Given that *A. m. adansonii* is not part of HBeeID and is taxonomically closer to *A. m. scutellata,* it received this latter assignation at the Tier II level (Fig. 4). Thus, within its limitation, HBeeID identified *A. m. adansonii* samples to the closest available African comparison, that of *A. m. scutellata*. As HBeeID chose the closest match available, the *A. m. adansonii* samples were also identified with 100% accuracy. Of the ten specimens from South Africa classified as *A. m. capensis,* five were identified as *A. m. capensis* and five as *A. m. scutellata*, resulting in a 50% accuracy (Table 2). A hybrid zone exists between these two subspecies and the precise demarcation of this boundary is likely fluid making it difficult to ascertain the identity samples from this region [101, 102].

The 39 samples in Harpur et al. [52] had 258 SNPs (95%) that overlap with the 272 in the HBeeID SNP panel. In addition, some samples had missing genotypes that ranged from 14 to 24 (5 to 9%). Of the ten *A. m. jemenitica* Ruttner, 1976 samples, HBeeID identified nine as America-Africanized and one as *A. m. scutellata.* Similarly, to *A. m. adansonii* mentioned above, *A. m. jemenitica* is not built into the reference base of HBeeID. Thus, the tool will assign samples of this subspecies to the closest available reference, namely *A. m. scutellata.* Of the eleven *A. m. scutellata* samples, nine were identified as *A. m. scutellata* and two as America-Africanized. The nine samples of *A. m. carnica* were identified to the closest available category in HBeeID, namely*,* as EU_Lig_like. The four samples from Spain were correctly identified as *A. m. iberiensis.* The five samples from Poland, all listed as belonging to the M lineage*,* were identified by HBeeID as follows: sample 218 (0.940% purity, levels from Wallberg et al. [53] as EU_Lig_like; sample 207 (0.999%. purity) as *A. m. iberiensis*, samples 226 and 217 (0.999% purity) as Puerto Rico and sample 227 (0.932% purity) also as Puerto Rico. HBeeID recognized the similarity of sample 207 to *A. m. iberiensis,* while for the remaining, which had from 15 to 18 missing SNPs, it assigned to the closest available match of EU_Lig_like and Puerto Rico. The total percentage match for the samples from Poland was 20%.

Of the 125 *A. m. mellifera* samples from Canada from Harpur et al. [88] 124 were correctly identified as EU_Lig_like and one sample was identified as America-Africanized. The HBeeID prediction accuracy for these samples was 99% (Table 2).

## Conclusion

The work presented herein demonstrates that selected, sparse genome information, as low as one in one million, can be used to assign individuals to populations effectively. HBs genotyped using the 272 SNP, Fluidigm-based assay, can differentiate unknown HBs from Africa, America, and Europe with a high degree of accuracy. This was demonstrated via a PCA analysis and genetic assignations obtained using the software program GeneClass2. Furthermore, 272 SNP-based genotype data from the 874 test HBs were used together with a hierarchical clustering method to delineate groups with similar genotype profiles, and these in turn used to develop a knowledge base network that formed HBeeID.

The evaluation of the HBeeID SNP diagnostic tool using one in-house and six publicly available data sets demonstrates that HBeeID is robust in its prediction of HB sample origin even when a large percentage of SNPs are missing (*ca.* 25%) in the unknown sample being tested. HBeeID can predict samples of pure and nearly pure European origin that are part of its reference base with a high degree of accuracy (> 95%). The tool also has a very robust capacity to predict samples that originate from Puerto Rico (near 100%) and a good capacity (90%) to discriminate HB samples with Africanized or African ancestry. Its prediction accuracy decreases when used to assess highly mixed individuals and populations represented by few individuals or not represented in the reference database.

The samples from the seven data sets used to test HBeeID only had a subset of the 272 SNPs that comprise the tool. Thus, the capacity of HBeeID to correctly assign ancestry to unknown samples, despite these limitations is remarkable. The prediction capacity of the HBeeID tool can be continuously improved by adding genotype data of samples tested. In addition, widening the geographic provenance of samples, and increasing the number of SNPs in HBeeID would further increase its predictive capacity and resolution. To develop the current HBeeID tool samples were obtained from a wider geographic area (*i.e.,* Americas, Africa, Europe, Eurasia) than previous SNP based attempts to discriminate HBs in world geographic areas, e.g., Eurasia [54], Europe [71, 72, 81, 82], Canada [83], South Africa [58].

### Future directions

Whole genome sequencing of HBs is costly and time consuming. In comparison, the screening of a HB sample using HBeeID’s 272 SNP-based assay is several orders of magnitude less. As a result, HBeeID will be of benefit to applied fields such as agriculture, pollination, conservation, public health and beekeeping and breeding. HBeeID could be used for stock confirmation in queen production as well as effectively utilized to track accidental HBs that are part of commercial goods at border crossings [22]. Moreover, HBeeID could be a valuable tool to assess fluctuations in the genetic diversity of populations as they adapt to environmental conditions due to climate change and detect species that are threatened [17]. The methodology used to develop HBeeID could also be used as template for tools for other organisms of ecological and economic importance. The ongoing increase in genomic data collection for bees and other organisms [103, 104] permits the development and makes it possible to improve the resolution of tools such as HBeeID. Increasing the number and geographic distribution of samples used as reference will extend HBeeID capacity to identify other subspecies of HBs within the O, M, C and Y lineages. In turn, HBeeID and similar tools can help to harness and utilize the ever-increasing genomic data.

### Supplementary Information

**Additional file 1.** List of Cooperators in this study (Table S1). List of honey bee samples used with the Fluidigm platform (Table S2)  and Agena (Table S3) and their respective locality of origin.

**Additional file 2.** Supplementary Methods 1.

**Additional file 3.** Fluidigm primers (Table S4) and Agena primers (Table S5).

**Additional file 4.** Genotype information for the 874 reference samples genotyped with Fluidigm along with their collection locations. (Table S6).  Genotype information, per Continent, Country and Region, for the 874 reference samples genotyped with Fluidigm (Table S7). A VCF file of this genotype data is also available at the European Variation Archive under accession # PRJEB74317.

**Additional file 5.** Interactive three-dimensional PCA plot showing the genotypic relationship of 874 HB samples genotyped using 272 SNPs using the Fluidigm genotyping platform (html based interface document).

**Additional file 6.** SNP genotype data used as input to test HBeeID. Data from this work and from published datasets: Data for this work; Cridland et al. [57]; Avalos et al. [84]; Kadri et al. [93]; Wallberg et al. [53]; Harpur et al. [52]; Harpur et al. [88] (Table S8).

**Additional file 7.** Results for the 874 reference samples of HBeeID analyzed using GeneClass2 (GC2). Reference Sets I, II, III and geolocation map (Table S9).

**Additional file 8.**  SNP genotypes for all the 874 test samples genotyped using the Fluidigm platform in genepop format for 251 SNPs that are part of the 272 SNP panel that are common between the SNP datasets from Avalos et al. [84] and Wallberg et al. [53] (Table S10).

**Additional file 9.** Reference set I genotype data used for GeneClass2 assignations (Table S11).

**Additional file 10.** Reference set II genotype data used for GeneClass2 assignations (Table S12).

**Additional file 11.** Reference set III genotype data used for GeneClass2 assignations (Table S13)

**Additional file 12.** HBeeID assignations of samples from the data sets used to test BeeID: Data from this work and published data sets: Cridland et al. [57]; Avalos et al. [84]; Kadri et al. [93]; Wallberg et al. [53]; Harpur et al. [52]; Harpur et al. [88] along with their metadata (Table S14).

## Data Availability

All data used to develop and test the HBeeID tool can be found in Supplementary Materials. The HBeeID tool along with scripts, supporting information and data to run it is on GitHub https://github.com/taoyudong/HBeeID. The variant data for this study have been deposited in the European Variation Archive (EVA) [105] at EMBL-EBI under accession number PRJEB74317 (https://www.ebi.ac.uk/eva/?eva-study=PRJEB74317). Project name: HBeeID. Operating system(s): Platform independent (Mac, Windows, Linux). Programming language: Python and Javascript. Other requirements: NA. License: GNU GPL. Any restrictions to use by non-academics No restrictions.

## Abbreviations

aCGH: Microarray based comparative genomic hybridization
DAPC: Discriminant analysis of principle components
HBs: Honey bees
HAC: Hierarchical agglomerative clustering
HBRC: Honey bee research community
NGS: Next generation sequencing
SNP: Single nucleotide polymorphism
PCA: Principal component analysis
RAPD: Random amplified polymorphic DNA
RFLP: Restriction fragment length polymorphism
