# Supplementary material for: HBeeID: a molecular tool that identifies honey bee subspecies from different geographic populations
Source: BMC Bioinformatics. 2024 Aug 27;25:278. doi: 10.1186/s12859-024-05776-9 (PMC11348773; doi:10.1186/s12859-024-05776-9)
Supplement: Supplementary file 2 — Additional file 2. Supplementary Methods 1. [file 12859_2024_5776_MOESM2_ESM.docx]

Title: HBeeID: A molecular tool that identifies honey bee subspecies from different geographic populations.

**Supplementary Methods 1**

***a. Processing of honey bee samples.***

All collaborators received a collection protocol to be followed to secure honey bee specimens.

All specimens were collected live and placed in 95% ETOH. Samples that arrived from other locations were transferred upon receipt to new 2 ml screw cap tubes, with fresh 95% ETOH, and checked for integrity and quality (e.g., ETOH immersion, mechanical damage, etc.), labeled as per their origin and stored at -20°C. Specimens from Puerto Rico were collected live with a plastic Ziploc bag, and immediately placed in fresh 95% ETOH 25ml Falcon tubes and then transferred to 2 ml screwcap tubes. Honey bees from Puerto Rico were collected at a minimum distance of 2 miles from each other to ensure capturing colony diversity, and geo-referenced. This distance (2 miles) is twice the average distance a nectar forager is travels from the hive, and three times the average distance for a pollen forager, e.g. (1). A workflow that illustrates an overview of the process undertaken to develop the HBeeID tool is illustrated in Fig. 2.

***b. DNA extraction and isolation protocol.***

Individual HB specimens were dissected using a stereomicroscope. A vertical cut was made along the middle of the thorax. Half of the thoracic muscles was retrieved with a sterile scalpel and needle which along with the entire head was used for the DNA extraction. All dissecting tools were sterilized between dissections. Samples were allowed to air dry to eliminate residual ETOH prior to DNA extraction. To extract DNA, the EZ1 DNA Investigator Kit (Qiagen, Valencia, CA, USA) was used according to manufacturer’s instructions. A brief overview of the protocol is as follows: Tissue was lysed in 10 µl Proteinase K and 190 µl Buffer G2, in 2 ml Safe-Lock microtubes. Tissue was homogenized by incorporating a 5 mm Ø sterile stainless-steel bead (Qiagen, Valencia, CA, USA) into the Safe-Lock microtubes, and assembled into a Tissue Lyser II (Qiagen, Valencia, CA, USA) operating at an oscillation frequency of 30 Hz for 15s. Subsequently, tubes were briefly centrifuged and incubated overnight in a water bath set at 56°C. Undissolved material was removed by centrifugation at 300 x g for 1 min, and clear lysate supernatant was then transferred into a 2 ml sample tube. Sample tubes were placed into an automated genomic DNA purification robot (EZ1® Advanced XL Robot, QIAGEN, Valencia, CA, USA), following the manufacturer’s protocol for isolation of Genomic DNA from Tissue and the EZ1 DNA Investigator Kit (Qiagen, Valencia, CA, USA). Total DNA was eluted in a volume of 50 µl. DNA concentrations were measured using the Qubit™ 3.0 Quantitation Fluorometer (Invitrogen™), following the manufacturer’s protocols. The calibration of the fluorometer was done using the Invitrogen™ Qubit™ 1X dsDNA HS Assay Lambda Standard. After measurement, total DNA was stored at -20°C prior to sequencing.

***c. Development of Agena SNP panel.***

For the development of the Agena assay we identified an initial set of 160 SNPs that could differentiate the 15 populations of interest. The Agena Biosciences Assay Design Suite v2.0 (ADS 2.0) was used to design PCR primers and single base extension probes for the 160 targets. Context sequences, annotated for proximal SNPs with 600 bp of flanking sequence on either side of the target SNP, were used as input into ADS 2.0. IPLEX. Default settings were used in all cases except for increasing the maximum amplicon length to 300 bp to avoid proximal SNPs and low complexity sequences. A total of four multiplexes were designed including two (40-plexes), one (30-plex) and one (50-plex). These SNP targets had been previously apportioned into subgroups pertaining to specific functions: for identifying PR bees (two 40-plexes), Africanized bees (one 30-plex), and European bees (one 50-plex), thus ADS 2.0 was run several times to facilitate these target groupings. Any targets that were rejected within each group were replaced with suitable alternate targets and a new design was initiated. After an initial round of testing, several of the SNP assays failed to produce results. For some of these targets PCR primers were redesigned using Primer-BLAST (NCBI). One multiplex had a high failure rate thus alternate SNP targets were selected. A new multiplex was designed using ADS 2.0 that included these alternate targets as well as alternates for failing SNPs from other wells. At the completion of the process, the four multiplexes consisted of 132 SNPs. Primer sequences designed by Agena for these SNPs are found in Suppl. Table S5. These comprised the Agena panel and were used to genotype 908 individual samples collected from Europe, Africa, and North and South America (Fig. 1). The metadata for these samples is given in Suppl. Table S3**.**

***c. Instructions on how to run HBeeID and format input files.***

**1_Required R packages**

Adegenet

Readxl

Openxlsx

Tidyverse

Readr

**2_Diagram of Workflow**


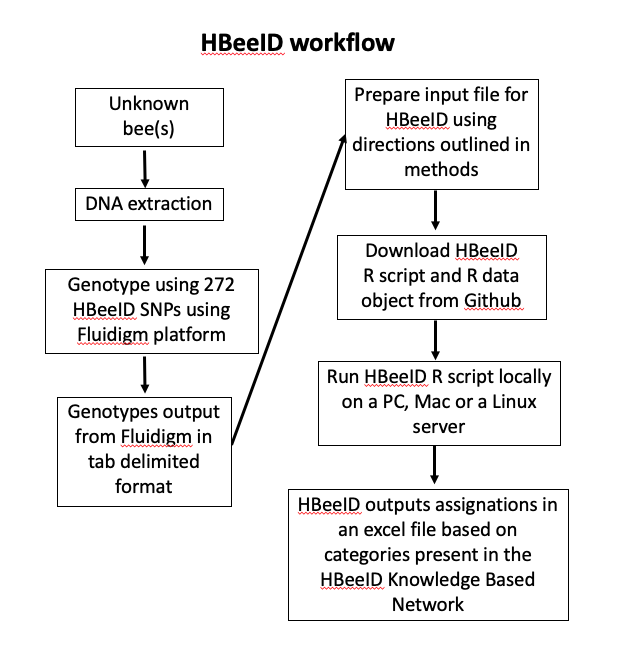


Figure S1. Diagram of workflow for HBeeID, A molecular tool that identifies honey bee subspecies from different geographic populations

***d. How to create the input file.***

HBeeID requires as input genotype data in CSV format where the sample identification codes are represented in rows and the SNP genotypes are shown in columns. The first row should contain headers that describe the content of each column.

The first column should contain sample identification codes. There should be a total of 273 columns, where the first column is the sample identification codes column, followed by the SNP genotypes for the 272 SNPs in HBeeID.

The file, [SNPs_272_IDs_order.xlsx], contains the list of the IDs for the 272 SNPs.

Each SNP ID contains the information of scaffold ID and the coordinate of the SNP loci in the *Apis mellifera* genome assembly, Amel4.5, available on BeeBase. The order of the SNPs in the input file must match the order shown in the file [SNPs_272_IDs_order.xlsx].

The genotype data should be converted to [0, 1 or 2] where [0] represents the homozygote state for the reference allele, [1] represents the heterozygote state and [2] represents the homozygote state for the alternate allele.

The input file, prepared as specified above, needs to be given as input under the following line within the BeeID code [unknowns_data <- read_csv("Input_genotypes.csv")].

To facilitate easy testing of the HBeeID the following test datasets are provided with data extracted in the required input format of HBeeID as described above.

1. Data from this work – 34 samples from PR
2. Cridland *et* al. (2) – 26 samples from Northern California and 18 samples from the Southern California
3. Avalos *et* al. (3) – 30 EHB samples from Hawaii; 28 AHB samples from Mexico; 30 PRHB samples from Puerto Rico
4. Kadri *et* al. (4) – 26 samples from Brazil
5. Wallberg *et* al. (5) – 10 *A. m. adansonii* samples, 10 Africanized samples from Brazil, 10 *A. m. anatoliaca* samples, 20 *A. m. mellifera* EU domestic, 10 *A. m. mellifera* US domestic, 10 *A. m. carnica* samples, 10 *A. m. capensis* samples, 10 *A. m. Iberiensis* samples, 10 *A. m. ligustica* samples, 20 *A. m. mellifera* Swedish-Norway samples, 10 *A. m. scutellata* samples.

***e. NCBI Accessions for data sets used to assess BeeID:***

Avalos *et al.* (3) NCBI BioProject PRJNA381313 at: <https://www.ncbi.nlm.nih.gov/search/all/?term=PRJNA381313>

Cridland et al. (2) NCBI BioProject PRJNA385500 at:

<https://www.ncbi.nlm.nih.gov/search/all/?term=PRJNA385500>

Kadri et al. (4) NCBI BioProject PRJNA324081 at:

<https://www.ncbi.nlm.nih.gov/search/all/?term=PRJNA324081>

Wallberg et al. (5) NCBI BioProject PRJNA236426 at:

<https://www.ncbi.nlm.nih.gov/search/all/?term=PRJNA236426>

***f. Script to convert haploid genotypes in VCF format to phased diplotized genotypes in VCF format.***

## This script is designed to take as input .vcf and output diplotized .vcf # ## file

#usr/bin/perl -w

$infile1 = $ARGV[0]; # .vcf file

$outfile = $ARGV[1]; # output file name

# Read .vcf.gz file

#open(READ, "gunzip -c $infile1 |") || die "cannot open pipe to $infile1";

open(READ, "<$infile1") or die "cannot open the file $infile1\n";

open(WR,">$outfile");

my @temp=();

my @temp2=();

my $id=();

my $line = ();

my %coords_hash=();

my %vals_hash=();

my @sampleids = ();

my @snpids = ();

while($line=<READ>) # read line by line .vcf.gz file

{

chomp($line);

if($line =~ /^\#CHROM/) # line that contains all sample ids

{

print WR "$line\n";

#@temp = split(/\t/,$line);

#@sampleids = @temp[9..@temp]; #print "@sampleids\n";

}

elsif($line !~ /^\#/) # SNP lines

{

#print "INside $line\n";

@temp = split(/\t/,$line);

$chr = $temp[0];

$crd = $temp[1];

for($i=9; $i <@temp; $i++)

{

# capture the (genotype)value just before the semicolon

($gtype) = ($temp[$i] =~ /(.*?)\:.*/);

# replace the existing value with the phased diploid genotype

$temp[$i] = $gtype."|".$gtype;

}

$line2 = join("\t", @temp);

print WR "$line2\n";

}

else # print the header

{

print WR "$line\n";

}

}

***g. Script to generate the proportional graphs in Figure 7 based on genotype data in*** ***Additional file 5: Supplementary Table***

## This script plots genotype dosage for

## selected terminal nodes in the AHB SNP Panel

#----------------------------------------------------------------------------

# Load analysis options and libraries

#----------------------------------------------------------------------------

# libraries

pkgs <- c("tidyverse", "magrittr", "RColorBrewer", "data.table", "MASS", "car", "BiocParallel")

invisible(lapply(pkgs, library, character.only = T))

rm(pkgs); gc()

# options

options(stringsAsFactors = F, scipen = 9999)

set.seed(12345)

#----------------------------------------------------------------------------

# Data Structuring

#----------------------------------------------------------------------------

# read in the files

gt <- fread("./data_sup_tab4.csv", sep = ",", data.table = F)

# create an apply loop that consolidates proportion of genotypes

nt.prop <- tapply(X = 1:nrow(gt),

INDEX = as.factor(gt$KBN_code),

FUN = function(i, data){

tmp = data[i, ]

prop = data.frame(REF = colSums(tmp == 0) / nrow(tmp),

HET = colSums(tmp == 1) / nrow(tmp),

ALT = colSums(tmp == 2) / nrow(tmp))

},

data = as.matrix(gt[, -c(1:10)])

)

# plot each node

pdf(file = "./terminal_nodes.pdf", width = 6.5, height = 2)

lapply(1:length(nt.prop), function(i, data){

print(i)

barplot(t(data[[i]]), col = c("green4", "grey80", "magenta3"),

border = NA, axes = F, xaxt = "n", ylab = names(data)[i])

}, data = nt.prop)

dev.off()

# repeat this process for the head nodes

# create the index

h.idx <- setNames(

nm = unique(gt$KBN_code),

object = c("A_2", "A_1", "A_2", "A_2", "A_1", "A_1", "A_1", "A_1", "A_2", "A_2")

)

#apply the same summary to the head nodes

h.prop <- tapply(X = 1:nrow(gt),

INDEX = as.factor(as.character(h.idx[gt$KBN_code])),

FUN = function(i, data){

tmp = data[i, ]

prop = data.frame(REF = colSums(tmp == 0) / nrow(tmp),

HET = colSums(tmp == 1) / nrow(tmp),

ALT = colSums(tmp == 2) / nrow(tmp))

},

data = as.matrix(gt[, -c(1:10)])

)

# plot the head nodes

pdf(file = "./head_nodes.pdf", width = 6.5, height = 2)

lapply(1:length(h.prop), function(i, data){

print(i)

barplot(t(data[[i]]), col = c("green4", "grey80", "magenta3"),

border = NA, axes = F, xaxt = "n", ylab = names(data)[i])

}, data = h.prop)

dev.off()

**References**

1. Couvillon MJ, Schürch R, Ratnieks FLW. Waggle Dance Distances as Integrative Indicators of Seasonal Foraging Challenges. PLoS One, 9 (2014): p. e93495

2. Cridland JM, Ramirez SR, Dean CA, Sciligo A, Tsutsui ND. Genome Sequencing of Museum Specimens Reveals Rapid Changes in the Genetic Composition of Honey Bees in California. Genome Biol Evol. 2018 Feb 1;10(2):458–72.

3. Avalos A, Pan H, Li C, Acevedo-Gonzalez JP, Rendon G, Fields CJ, et al. A soft selective sweep during rapid evolution of gentle behaviour in an Africanized honeybee. Nat Commun. 2017 Nov 16;8(1):1550.

4. Kadri SM, Harpur BA, Orsi RO, Zayed A. A variant reference data set for the Africanized honeybee, Apis mellifera. Sci Data. 2016 Nov 8;3(1):160097.

5. Wallberg A, Han F, Wellhagen G, Dahle B, Kawata M, Haddad N, et al. A worldwide survey of genome sequence variation provides insight into the evolutionary history of the honeybee Apis mellifera. Nat Genet. 2014 Oct;46(10):1081–8.
