# Supplementary material for: HBeeID: a molecular tool that identifies honey bee subspecies from different geographic populations
Source: BMC Bioinformatics. 2024 Aug 27;25:278. doi: 10.1186/s12859-024-05776-9 (PMC11348773; doi:10.1186/s12859-024-05776-9)
Supplement: Supplementary file 5 — Additional file 5. Interactive three-dimensional PCA plot showing the genotypic relationship of 874 HB samples genotyped using 272 SNPs using the Fluidigm genotyping platform (html based interface document). [file 12859_2024_5776_MOESM5_ESM.html]

HBeeID Interactive Data Visualization


# HBeeID: Interactive Data Visualization

Interactive 3D version of Fig. 5 in the paper titled "HBeeID: A molecular tool that identifies honey bee subspecies from different geographic populations."
Colored circles in the PCA plot represent the 874 honey bee reference samples genotyped with the 272 SNPs via the Fluidigm genotyping platform that constitute HBeeID.

### Interaction Instructions:

(1) To determine the geographic origin of a sample in the PCA plot click on one of the circles, subsequently a red circle will appear in the map corresponding to the location of the sample while all the other samples on the map will fade. (A short delay might be expected after selecting a point)  
(2) When a given circle in the PCA plot is selected, information will appear at the bottom of the PCA plot indicating the collection information for that sample.   
(3) To select a new point, click the new point of interest in the plot.  
(4) To revert to none point being selected, click the currently selected point again in the plot or on the map. (A short delay might be expected after unselecting a point)  
(5) All points of a single country can be hidden/displayed in the plot and the map by clicking the country name or corresponding point in the legend.  
(6) To more easily view samples from a given country that overlap with others the viewer can select all countries to remove all points and then repopulate the plot to view samples from a single or multiple countries.  
(7) Refreshing the page will reset the plot.

### 3D PCA Scatter Plot

### Geolocation of Samples

### Info of the Selected Point:

No point has been selected yet
